# Genetically modified pigs are protected from classical swine fever virus

**DOI:** 10.1371/journal.ppat.1007193

**Published:** 2018-12-13

**Authors:** Zicong Xie, Daxin Pang, Hongming Yuan, Huping Jiao, Chao Lu, Kankan Wang, Qiangbing Yang, Mengjing Li, Xue Chen, Tingting Yu, Xinrong Chen, Zhen Dai, Yani Peng, Xiaochun Tang, Zhanjun Li, Tiedong Wang, Huancheng Guo, Li Li, Changchun Tu, Liangxue Lai, Hongsheng Ouyang

**Affiliations:** 1 Jilin Provincial Key Laboratory of Animal Embryo Engineering, Institute of Zoonosis, College of Animal Sciences, Jilin University, 130062, Changchun, Jilin Province, People’s Republic of China; 2 Key Laboratory of Jilin Province for Zoonosis Prevention and Control, Institute of Military Veterinary, Academy of Military Medical Sciences, Changchun, Jilin Province, People’s Republic of China; University of California Riverside, UNITED STATES

## Abstract

Classical swine fever (CSF) caused by classical swine fever virus (CSFV) is one of the most detrimental diseases, and leads to significant economic losses in the swine industry. Despite efforts by many government authorities to stamp out the disease from national pig populations, the disease remains widespread. Here, antiviral small hairpin RNAs (shRNAs) were selected and then inserted at the porcine *Rosa26* (*pRosa26*) locus via a CRISPR/Cas9-mediated knock-in strategy. Finally, anti-CSFV transgenic (TG) pigs were produced by somatic nuclear transfer (SCNT). Notably, in vitro and in vivo viral challenge assays further demonstrated that these TG pigs could effectively limit the replication of CSFV and reduce CSFV-associated clinical signs and mortality, and disease resistance could be stably transmitted to the F1-generation. Altogether, our work demonstrated that RNA interference (RNAi) technology combining CRISPR/Cas9 technology offered the possibility to produce TG animal with improved resistance to viral infection. The use of these TG pigs can reduce CSF-related economic losses and this antiviral strategy may be useful for future antiviral research.

## Author summary

Classical swine fever (CSF), caused by classical swine fever virus (CSFV), and is a highly contagious, often fatal porcine disease that causes significant economic losses. Due to the economic importance of this virus to the pig industry, the biology and pathogenesis of CSFV have been investigated extensively. Despite efforts by many government authorities to stamp out the disease from national pig populations, the disease remains widespread, and it is only a matter of time before the virus is reintroduced and the next round of disease outbreaks occurs. These findings highlight the necessity and urgency for developing effective approaches to eradicate the challenging CSFV. In this study, we successfully produced anti-CSFV TG pigs by combining RNAi technology and CRISPR/Cas9 technologies, and viral challenge results confirmed that these TG pigs could effectively limit the replication of CSFV in vivo and in vitro. Additionally, we confirmed that the disease resistance traits in the TG founders were stably transmitted to their F1-generation offspring. Altogether, our work reported the combinational application of CRISPR/Cas9 and RNA interference (RNAi) technology in the generation of anti-CSFV TG pigs, it provided an alternative strategy to change the virus. The results of this study suggested that these TG pigs offered potential benefits over commercial vaccination and reduced CSFV-related economic losses.

## Introduction

Classical swine fever virus (CSFV) belongs to the genus Pestivirus within the family Flaviviridae [1]. CSFV is an enveloped virus that possesses a single-strand positive-sense 12. 3kb RNA genome, which contains a long open reading frame that encodes a 3898-amino acid (aa) poly-protein [2]. The co- and post-translational processing of the poly-protein by cellular and viral proteases results in cleavage of the poly-protein into individual proteins [3], including four structural proteins (C, Erns, E1 and E2) and eight non-structural proteins (Npro, p7, NS2, NS3, NS4A, NS4B, NS5A and NS5B) [4].

Classical swine fever (CSF) has tremendous impact on animal health and the pig industry. CSFV can be transmitted both horizontally and vertically, and domestic pigs and wild boar are highly susceptible to CSFV infection. CSFV can cause severe leukopenia and immunosuppression, which often leads to secondary enteric or respiratory infections [5]. Congenital infection with CSFV can result in persistently infected animals, which do not develop specific antibodies against the virus [6]. This effect is probably due to immunotolerance developed during foetal lymphocyte differentiation. Persistently infected animals continuously shed the virus and are potential sources of new CSF outbreaks, in addition, this phenomenon leads to difficulties in diagnosis[7]. Infections with highly virulent CSFV strains exhibit low age dependence of clinical courses and may result in 100% mortality in all age classes of animals and severe neurological signs within 7 to 10 days [8]. The economic losses caused by an outbreak in the Netherlands in 1997 were as high as 2.3 billion USD, and more than 11 million pigs had to be destroyed. Pigs are infected with CSFV strains that were recently in circulation in Europe became severely ill, and up to 90% died within 4 weeks after infection [9]. Additionally, infected pork and pork products are dangerous sources of CSFV.

Strategies to control CSFV mainly consist of a systematic prophylactic vaccination policy and a non-vaccination stamping-out policy [10]. In 2016, 22 countries officially reported mandatory vaccination campaigns (OIE WAHIS). Compulsory vaccination is the current policy in China, and vaccination coverage must be greater than 90% at any time of the year in the swine population [11]. Despite efforts by many government authorities to stamp out the disease from national pig populations, the disease remains widespread in several countries of South and Central America and parts of Eastern Europe and neighbouring countries, as well as Asia, including India, and parts of Africa [10,12]. It is only a matter of time before the virus is reintroduced and the next round of disease outbreaks occurs. These findings highlight the necessity and urgency for developing highly effective approaches to eradicate the challenging CSFV. An alternative strategy is to develop TG pigs that are genetically resistant to CSFV infection.

The rapid development of genome editing technologies has facilitated studies that explore specific gene functions and the establishment of animal models. The production of genetically modified animals with viral resistance by the versatile CRISPR/Cas9 system has been recently examined by several researchers [13,14]. In livestock, these technologies have contributed to the development of virus-resistant animals and have provided considerable productivity benefits to producers.

RNAi is a natural post-transcriptional gene silencing mechanism [15], and since its discovery, RNAi has been regarded by virologists as a promising method for the suppression of viral infection[16–19]. Previous studies have shown that miR30-based shRNA design is suitable due to the potency of these shRNAs and the ease of expressing shRNA from a variety of promoters [20,21]. *Rosa26* is ubiquitously expressed in embryonic and adult tissues and was first identified and targeted in mouse embryonic stem cells (ESCs) in the 1990s[22]. Since the discovery of *Rosa26*, hundreds of TG animals and cell lines expressing a variety of transgenes have been successfully created using the *Rosa26* locus [23–26]. The sequence of the porcine *Rosa26* (*pRosa26*) locus has been completely characterized, and the *pRosa26* promoter has been identified [27,28]. In addition, our previous study confirmed that expression of site-specifically inserted EGFP can be strongly and consistently driven by the *pRosa26* promoter, similar to the mouse promoter[29], indicating that the porcine endogenous promoter is not rejected in the porcine cellular context by epigenetic silencing. To date, there have been several reported RNAi-based studies on CSFV suppression in vitro, and these studies have indicated that the development of shRNA-TG pigs that are resistant to CSFV may be possible.

Therefore, in this study, we combined CRISPR/Cas9 technology and RNAi technology to generate TG pigs with a knock-in of a defined antiviral shRNA, and then assessed the transgene resistance of these pigs to CSFV infection (S1 Fig).

## Results

### Selection of antiviral siRNAs and shRNA knock-in cell lines

**Fig 1:**
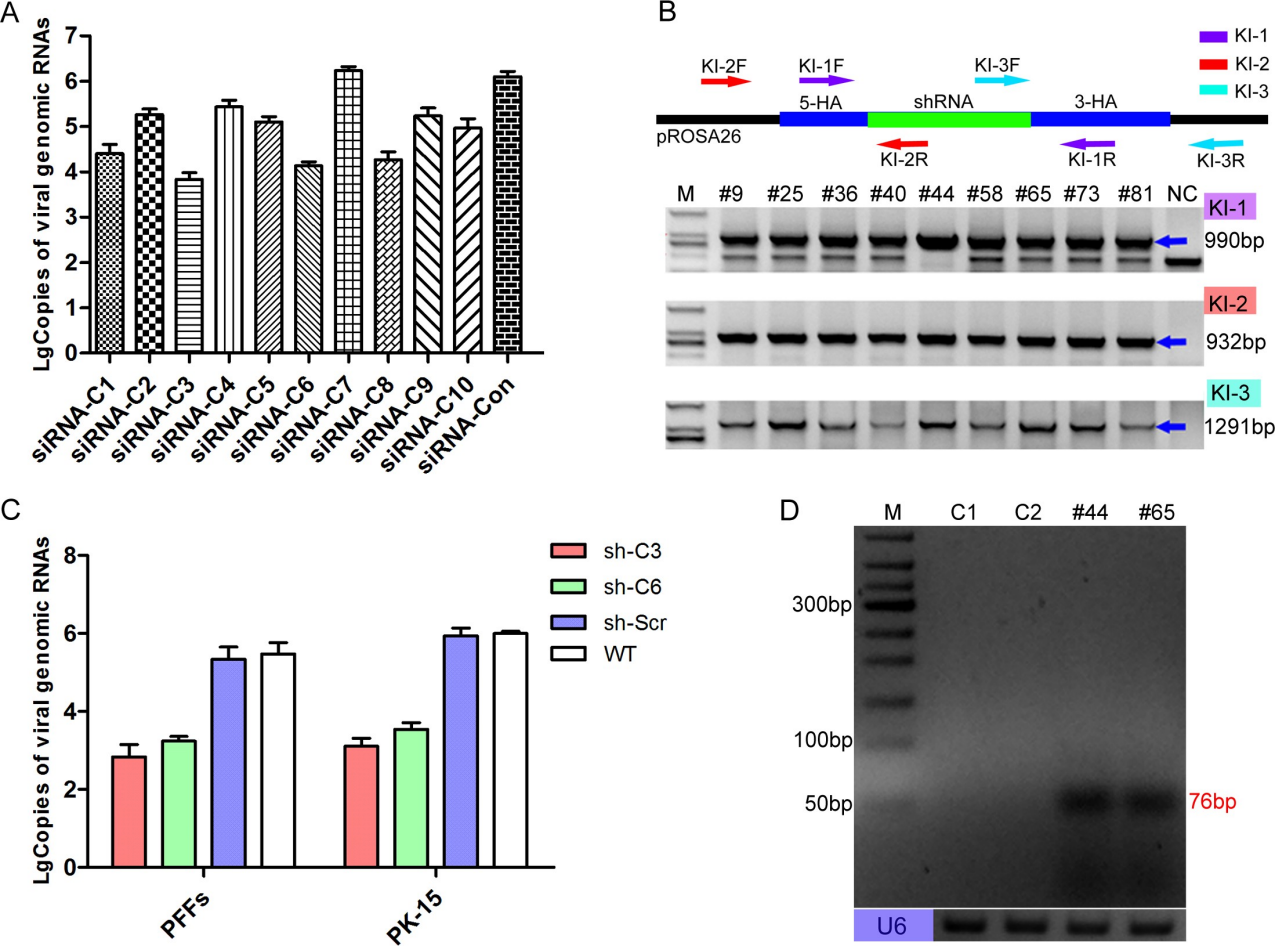
Selection of antiviral cell clones. (**A**) Reduction of viral genome replication in siRNA-transfected cells was further assessed by real time PCR at 72 h post-infection. Error bars represent the SEMs, n = 3. (**B**) Genomic PCR analysis confirmed the knock-in events at the p*Rosa26* locus by using specific primers (**Table 4**). The KI-1 primers were used to determine homozygosity or heterozygosity, the KI-2 primers amplified the 5’ junction, and the KI-3 primers amplified the 3’ junction junction. The blue arrows indicate target amplicons, and the corresponding sizes of the PCR amplicons are 990, 932 and 1291 bp. Lanes 2–10 represent the positive shRNA knock-in PFF clones. NC: negative control (wild-type PFFs). M: D2000. The corresponding sequences of these primers are listed in Table 4. (**C**) Inhibition of viable viral production in shRNA knock-in cell clones (PFF and PK-15 cell clones) was further assessed by real-time PCR at 72 h post-infection. The copies of the viral genomes were analysed using the unpaired t-test. Error bars represent the SEMs, n = 3. Sh-C3: sh-C3 knock-in cell clone. Sh-C6: sh-C6 knock-in cell clone. Sh-Scr: scrambled shRNA knock-in cell clone. WT: wild-type PFFs. (**D**) Expression of the two targeting siRNAs in the corresponding transgenic PFF clones was confirmed by RT-PCR. M: 50bp DNA ladder. C1: wild-type PFFs. C2: scrambled shRNA transgenic PFF clones. #44: shRNA-C3 transgenic PFF clones. #65: shRNA-C6 transgenic PFF clones. The size of the target amplicons was 76 bp. Endogenous U6 was used as an RNA quality and loading control.

We first aimed to select small interfering RNAs (siRNAs) that could efficiently inhibit CSFV. For potent and durable inhibition of viral replication, it is important to target viral sequences that are essential and well conserved among different viral strains to reduce the chance of escape [30,31]. Ten well-conserved siRNAs (Table 1) were designed and individually introduced into PK-15 cells by electroporation. At 72 h post-infection, the viral inhibition efficiencies of each siRNA were evaluated by qPCR (Fig 1A) and indirect immunofluorescence assay (IFA) (S2A Fig) [32–34]. Of these siRNAs, siRNA-C3 and siRNA-C6 exhibited highest antiviral activity (S2B Fig). Next, we determined whether CSFV could be inhibited in cells stably expressing siRNA-C3 and siRNA-C6. Finally, the two siRNAs were separately cloned into a miR30 backbone [35] and to finally obtain two single-shRNA-expressing targeting vectors (S3A Fig). The knock-in site was located in the first intron, and the endogenous p*Rosa26* promoter was utilized to express the antiviral shRNA gene (S3B Fig).

**Table 1:** siRNAs sequences.

Name of siRNA	Sense(5'-3')	Antisense(5'-3')
siRNA-C1	ACAGGUUACAGAAUAGUAGAU**tt**	AUCUACUAUUCUGUAACCUGU**tt**
siRNA-C2	CUCUCUGGUUGUUCUGUCUGA**tt**	UCAGACAGAACAACCAGAGAG**tt**
siRNA-C3	ACACUGUGACAGACUAUGUAA**tt**	UUACAUAGUCUGUCACAGUGU**tt**
siRNA-C4	CAGAGUCAGUAUACCAGUAUA**tt**	UAUACUGGUAUACUGACUCUG**tt**
siRNA-C5	CCGACUGAUUGAAUUGGUACA**tt**	UGUACCAAUUCAAUCAGUCGG**tt**
siRNA-C6	GAACCCUGAAGUGGAUUAGCC**tt**	GGCUAAUCCACUUCAGGGUUC**tt**
siRNA-C7	AGUGGCAACCAUCUAGGCCCGG**tt**	CCGGGCCUAGATGGUUGCCACU**tt**
siRNA-C8	CCUGUACAUUCAACUACGCAA**tt**	UUGCGUAGUUGAAUGUACAGG**tt**
siRNA-C9	ACCCGAGUUAGAGUCCUCCUA**tt**	UAGGAGGACUCUAACUCGGGU**tt**
siRNA-C10	GCCUACCAAUUUGAUGAUAUU**tt**	AAUAUCAUCAAAUUGGUAGGC**tt**
siRNA-Con	CCAUGUGAUUUUGUUGUUAAU**tt**	AUUAACAACAAAAUCACAUGG**tt**

Next, the two shRNA targeting vectors were separately electroporated into porcine foetal fibroblasts (PFFs) with the CRISPR/Cas9 vector; shRNA knock-in PFFs were selected with the limiting dilution method; and knock-in events were confirmed by PCR with specific primers and Sanger sequencing (Fig 1B and S3C Fig). Then, we inoculated these knock-in PFFs with CSFV, and 72 h post-infection, the antiviral activity of these knock-in PFF lines were examined by qPCR (Fig 1C) and IFA (S4A Fig). Similar to the experiments involving transiently transfected siRNAs, the viral challenge results indicated that these knock-in PFF lines could effectively inhibit CSFV compared with the control PFFs. Additionally, we selected shRNA knock-in PK-15 cells, which is another porcine cell line that is highly susceptible to CSFV infection. As expected, a viral challenge assay of these PK-15 clones showed antiviral activity comparable to that of the PFF clones (Fig 1C and S4B Fig). Finally, the expression of antiviral siRNAs was successfully detected by RT-PCR (Fig 1D) and further confirmed by sequencing (S4C Fig). Together, these results suggested distinct role for shRNA in the inhibition of CSFV replication in these shRNA knock-in cell clones.

Previous study reported that nonspecific effects of shRNA (structure, chemical modification, concentration, and cellular localization) could stimulate interferon (IFN) response, leading to adverse effects, including the inhibition of cell division and growth and eventually to apoptosis [36,37]. First, to investigate the effect of the shRNA on cell growth, we used the CCK8 assay to monitor the viability of the shRNA knock-in cell clones. Compared to NTG clones, the CCK8 results showed that knock-in of selected shRNA in TG clones did not induce significant adverse effects (S4D Fig). Next, we measured the interferons in these TG clones by qRT-PCR. The results showed that the expression levels of IFN-α, IFN-β, and IL-6 in the shRNA knock-in PFF clones were similar to those in the non-transgenic (NTG) PFF clones (S4E Fig). To further assess the potential toxicity caused by shRNA and the further feasibility of generating TG pigs via somatic cell nuclear transfer (SCNT), these two shRNA knock-in PFF lines were used as donor cells for SCNT[38]. The results indicated that the blastocyst development rates were similar between the TG group and wild-type (WT) group (Table 2). Together, these results strongly suggest that the antiviral effect in TG cells occurred via RNAi-mediated sequence-specific inhibition and these shRNA knock-in PFFs could be further used to generate anti-CSFV TG pigs via SCNT.

**Table 2:** Statistical results of the blastocyst development rate.

Repeat Times of SCNT	Type of Donor Cell	Total Nuclear Donor Cell Number	Blastocyst Number	Blastocyst Development Rate
**1**	TG	296	74	25.0%
	WT	304	72	23.7%
**2**	TG	311	74	23.8%
	WT	306	79	25.8%
**3**	TG	287	70	24.4%
	WT	293	73	24.9%

### Production of F0-generation TG pigs and verification of the antiviral ability of isolated TG cells

**Fig 2:**
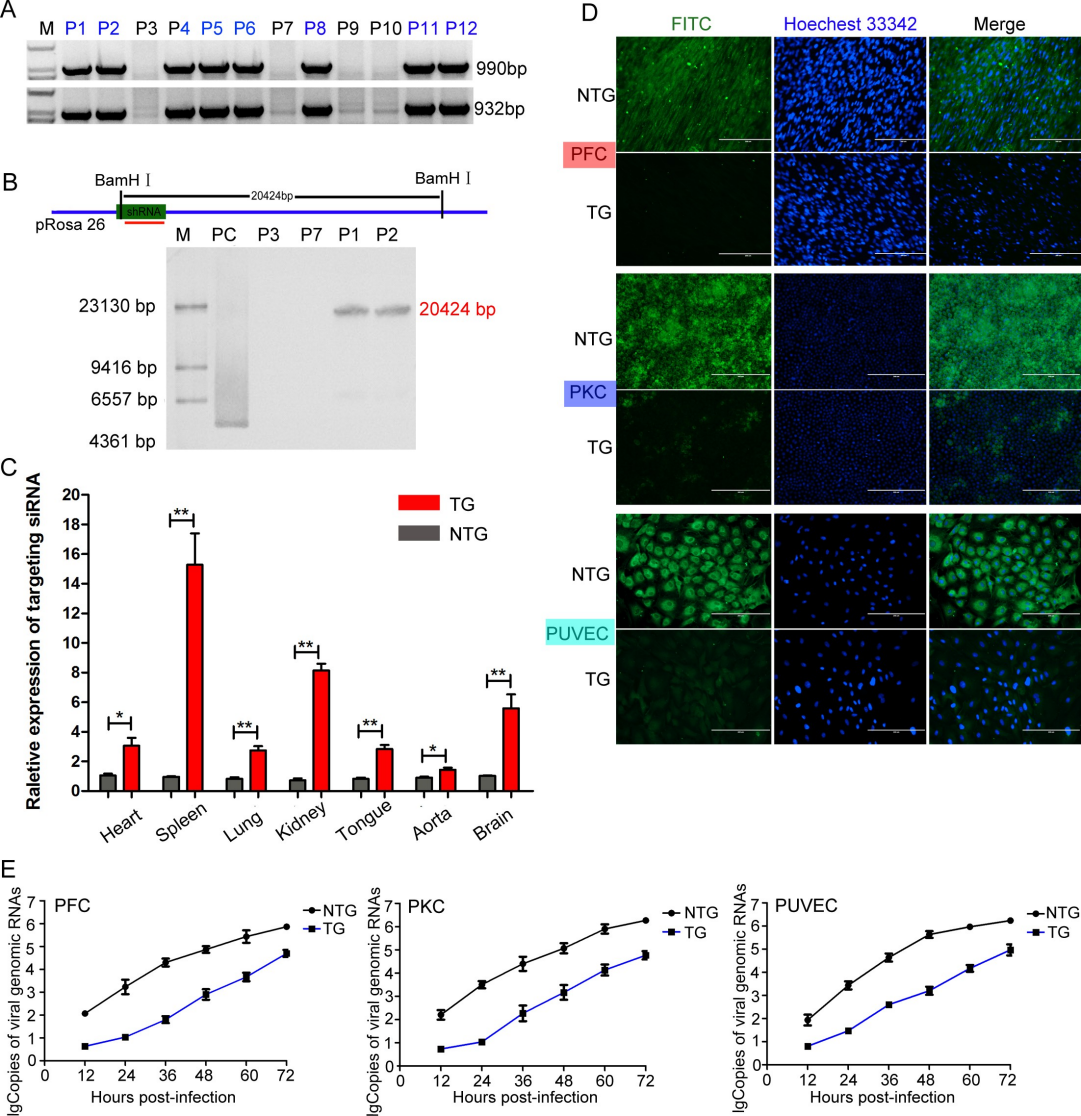
Generation of F0-generation TG pigs expressing the targeting siRNA. **(A**) Genomic PCR analysis of the F0-generation to identify TG pigs. P1, P2, P4, P5, P6, P8, P11 and P12 indicate TG pigs, and P3, P7, P9 and P10 indicate NTG pigs. M: D2000. (**B**) Schematic for Southern blotting. The hybridization signals indicated that the shRNA gene was successfully integrated into the p*Rosa26* locus in the TG pigs. M: DNA maker. PC: positive control plasmid. P3 and P7 indicate NTG pigs, and P1 and P2 indicate TG pigs. (**C**) The relative expression levels of the targeting siRNA in various tissues and organs from TG pigs were determined by qPCR. The expression levels were analysed using the unpaired t-test (*p<0.05; **p<0.01). Error bars represent the SEM, n = 3. TG: transgenic pigs. NTG: wild-type pigs. (**D**) Virus resistance in three types of isolated primary cells from TG and NTG pigs was examined by IFA. The cells cultured in 24-well plates were inoculated with 200 TCID_50_ of CSFV (Shimen strain). At 72 hours post infection, the CSFV-infected cells were incubated with an E2-specific antibody (PAb) and then stained with fluorescein isothiocyanate (FITC)-labelled goat anti-pig IgG (1:100). Cells were analysed under fluorescence microscope. PFC: porcine fibroblast cells. PUVEC: porcine umbilical vein endothelial cells. PKC: primary kidney cells. (**E**) Genomic replication kinetics of CSFV in challenged TG and NTG cells at various time points post-infection. Error bars represent the SEMs, n = 3.

Then, reconstructed embryos were transferred into surrogate sows, and 8 female TG pigs derived from the same PFF cell clone (shRNA-C3) were successfully obtained among 12 newborns (Fig 2A). Southern blotting (Fig 2B) and qPCR (S7A Fig.) experiments were further designed to investigate whether the shRNA gene was inserted at the pre-determined genome locus and to determine the copy numbers of the shRNA gene. As expected, the results confirmed the site-specific integration of antiviral shRNA in the TG pigs. Consistent with the expression pattern of the p*Rosa26* promoter [39,40], we further confirmed the relative expression level of the shRNA in various tissues, organs and cells in the TG pigs (Fig 2C and S5A Fig). To investigate whether the isolated TG cells from the TG pigs could effectively inhibit CSFV infection, three different types of primary cells were separately isolated from TG and NTG pigs (S5B Fig). Then, we inoculated these cells with CSFV, and 72 h post-infection, the results indicated that these isolated TG cells could effectively decrease CSFV infection compared to NTG cells, as shown in Fig 2D. Additionally, the replication characteristics of CSFV in the TG and NTG cells were evaluated via a multistep growth curve (Fig 2E).

Viral escape from RNAi-mediated inhibition has been described for several viruses [41–43]. To test whether the results shown in Fig 2D indicated viral escape, we further performed a viral escape study (S6A Fig). Then, target sequences were amplified by PCR with specific primer pairs (S6B Fig.), and the shRNA target sequence and flanking regions were further sequenced (S6C Fig). Sequencing results proved that there were no viral escape variants in these TG cells.

### Off-target analysis

**Fig 3:**
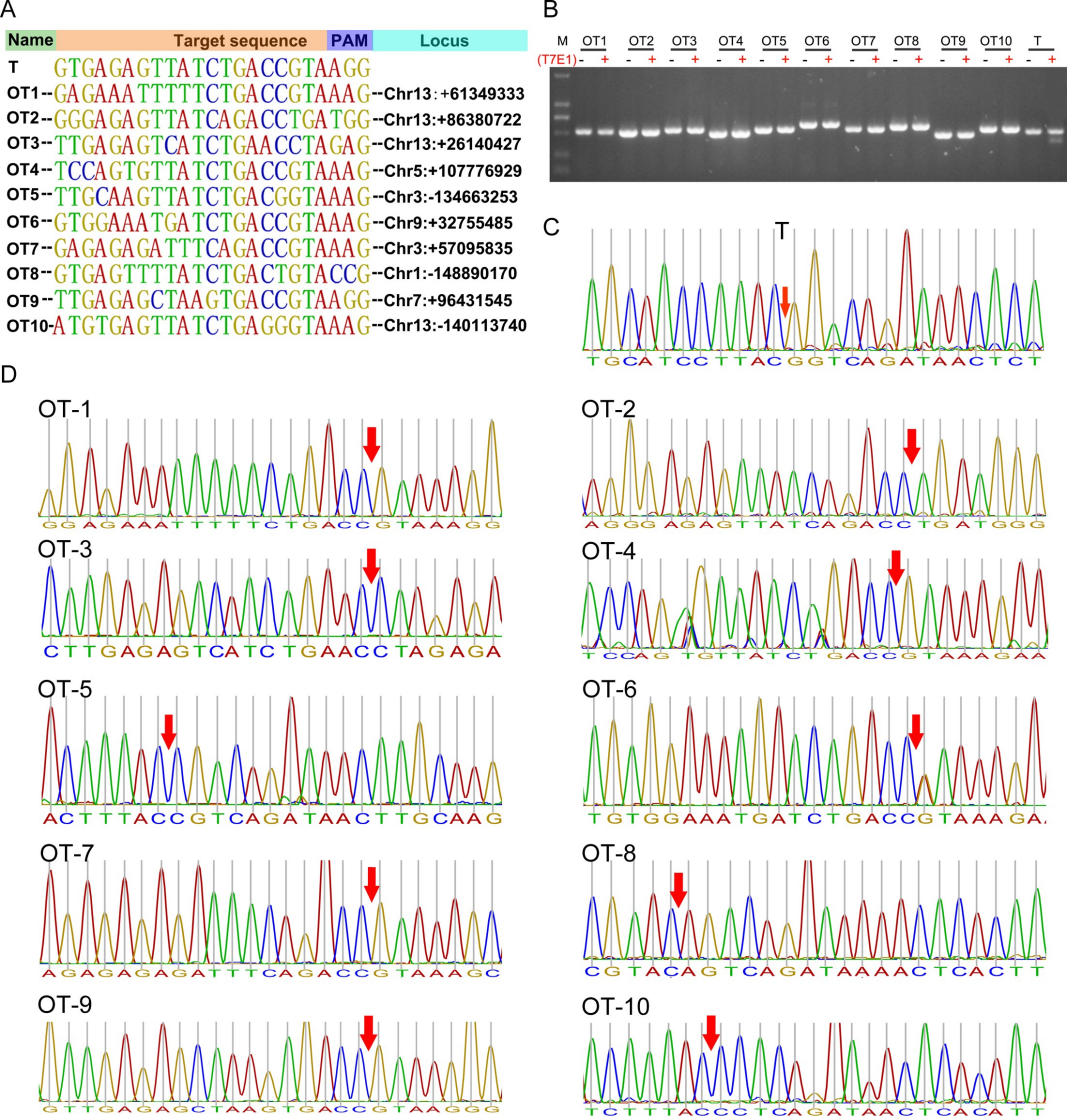
Off-target site analysis. (**A**) The target site (T) and ten predicted off-target sites (OT) for sgRNA91/Cas9. OT1~OT10 represents 10 potential off-target sites, and T represents target site. (**B**) T7E1 cleavage assays for the target site and the ten potential off-target sites; M, DL2000; (**C**) Sanger sequencing analyses of PCR amplicons that spanning the target sites. The cleavage sites are labeled with red arrow. (**D**) Sanger sequencing analyses of PCR amplicons that spanning the potential off-target sites. The potential cleavage sites are labeled with red arrow.

Potential off-target mutations can hinder with the application of the CRISPR/Cas9 system [13,14,44]. To test whether off-target were present in these genetically modified pigs; we screened the pig genome and predicted a total of 10 potential off-target sites (OTs) (Fig 3A). The fragments around all the potential off-target loci were amplified by PCR, and then subjected to T7E1 cleavage assay (Fig 3B). No mutation was detected in any locus. Consistent with the T7E1 results, further DNA sequencing results also showed no mutations at these potential off-target loci (Fig 3C and Fig 3D), suggesting that the CRISPR/Cas9 could be a reliable tool for targeting the genomes of genetically modified pigs.

### Generation of TG offspring and analysis of hereditary stability

**Fig 4:**
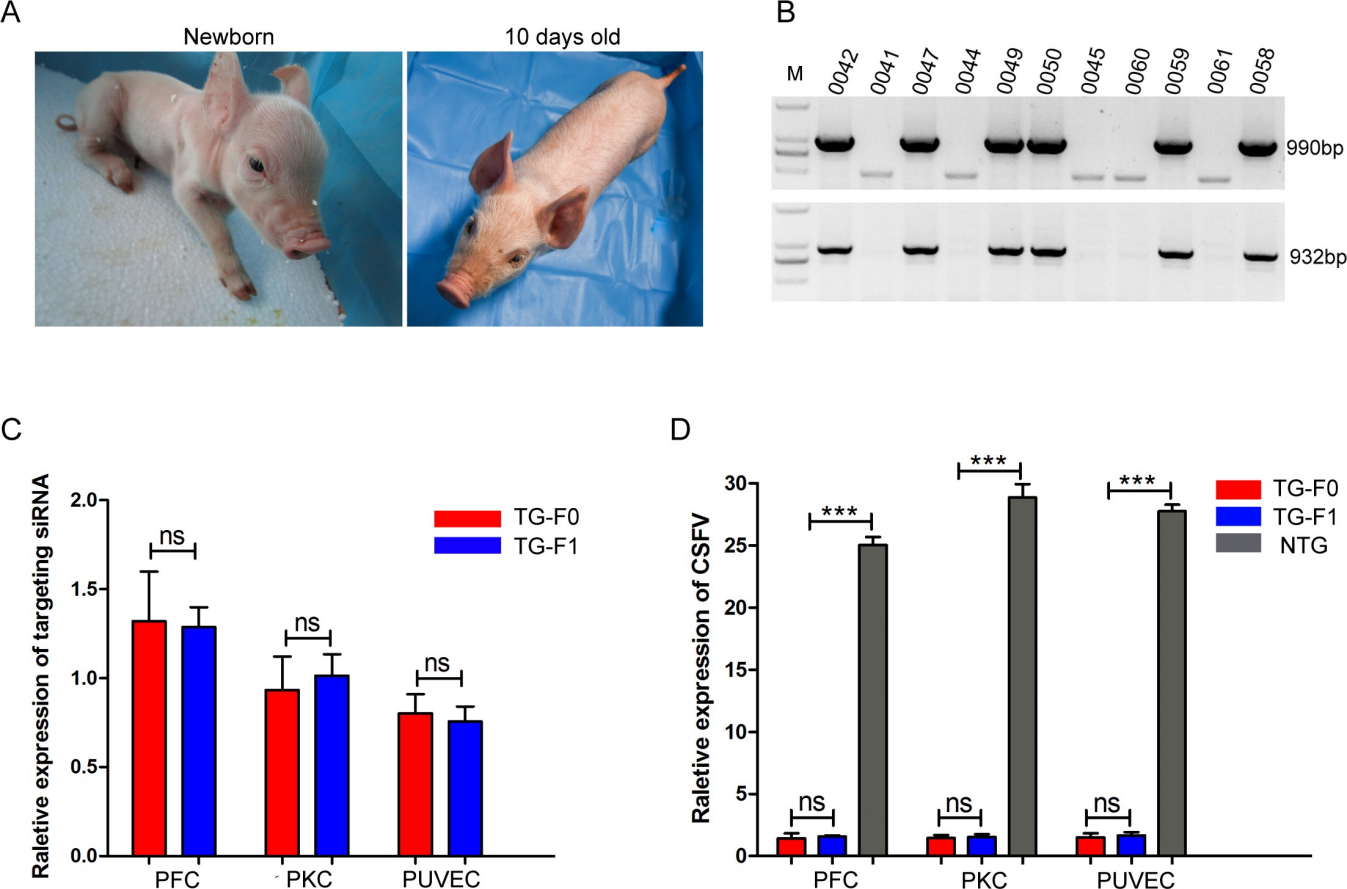
Generation of F1-generation TG pigs. **(A**) Photographs show one of the F1-generation TG piglets. (**B**) The knock-in events in newborn TG piglets were confirmed by genomic PCR. Pigs 0042, 0047, 0049, 0050, 0058 and 0059 are TG pigs, and pigs 0041, 0044, 0045, 0060 and 0061 were NTG. M, DNA marker (D2000, Tiangen, Beijing, China). (**C**) The relative expression levels of the targeting siRNA in F0- and F1-transgenic cells were compared by qPCR. Targeting siRNA expression levels were analysed with the unpaired t-test (ns. not significant). Error bars represent the SEMs, n = 3. TG-F1 represents F0-generation TG pigs. TG-F1 represents F1-generation TG pigs. (**D**) qPCR results show the relative expression of viral RNA in NTG cells, F0-generation TG cells and F1-generation TG cells. Viral RNA expression was analysed with an unpaired t-test (ns. not significant; ***p<0.001). Error bars represent the SEMs, n = 3.

When the TG founders were sexually mature, we obtained 11 F1-generation piglets by crossing the TG founders with WT pigs. Of these piglets, 6 were TG offspring and 5 were NTG offspring (Fig 4A and 4B). Compared with the littermate NTG piglets, these TG piglets exhibited a stable shRNA gene copy number (S7A Fig), normal porcine diploid chromosome number (2n = 38) (S7B Fig) and normal birth weight. Additionally, the sex and positive rate of the newborn piglets exhibited no deviation from the Mendelian law of inheritance. Interestingly, we also found that a molecular beacon could be used to identify differences in the expression levels of the targeting siRNA between the TG pigs and NTG pigs (S8 Fig) and that the expression pattern of the targeting siRNA in the F1-generation TG pigs was similar to that in their mother.

Importantly, we compared the expression level of the targeting siRNA between the TG founders and their TG offspring, and the results indicated that there was no significant difference in shRNA expression level between the F0- and F1-generation TG pigs (Fig 4C). Moreover, the viral challenge assay showed that the antiviral activity of the parental TG cells was similar to that of their filial TG cells (Fig 4D, S7C and S7D Fig). Together, these findings suggested that the TG pigs exhibited a greater inhibition of CSFV infection than the NTG pigs and that the RNAi-mediated antiviral activity could be stably transmitted to their offspring.

### TG pigs resisted CSFV infection

**Fig 5:**
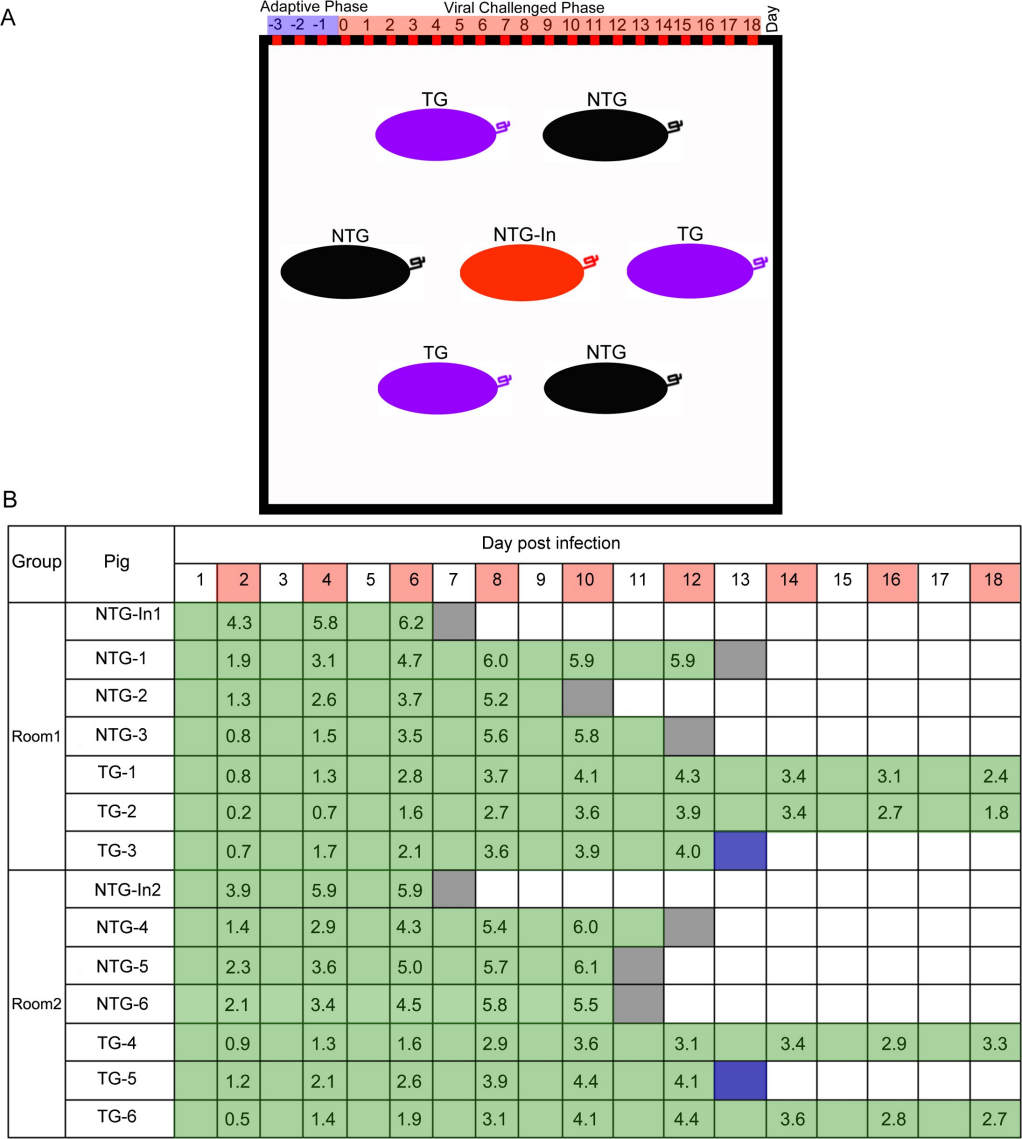
Experimental grouping and relative clinical monitoring data of the challenged pigs. (**A**) Schematic depiction of the in-contact challenge assay. The black box indicates a separate room. Different colours indicate different types of pigs. Each room included one non-TG pig that was injected with CSFV (NTG-In), three NTG pigs and three TG pigs. (**B**) Mortality and viremia in challenged pigs. The survival time of each pig is indicated by the length of the green bar. Red squares indicate the collection of blood samples. The terminal block colour indicates day and cause of death (black, found dead; blue, pigs (TG-4 and TG-5) euthanized for comparison to the dead NTG pig). The numbers for each day are the lg values of viral RNA copies present in the blood samples.

CSF is an acute and highly contagious disease in pigs. In most cases of natural infection, CSFV is mainly transmitted by direct contact with infected animals or by indirectly via infected faeces, food, or carcasses [9]. Therefore, we decided to perform animal challenge experiments with an “in-contact” infectious mode of infection. Next, all of the pigs were randomly distributed into two separate rooms, and each room included one non-TG pig that was used CSFV infection (NTG-In), three NTG pigs and three TG pigs (Fig 5A). Prior to viral challenge, all the pigs were tested for several common pig-associated viruses (Table 3) and CSFV specific antibodies. After three days of acclimation, these NTG-In pigs were directly challenged with CSFV by intramuscular injection, while the other housed pigs were not injected so that the natural spread of CSFV via cohabitation would be mimicked. During the course of infection, CSFV-associated clinical symptoms (including lethargy (S9A Fig) and haemorrhage (S9B Fig)), mortality and viremia (Fig 5B) in the challenged pigs were monitored and recorded daily. The results showed that all of the NTG pigs developed typical signs of CSFV infection, with a mortality rate of 100%. Although CSFV-associated clinical symptoms were also observed in the TG pigs, these symptoms were not serious, and the virus levels in the blood were lower in the TG pigs than in the NTG pigs.

**Table 3:** Detection of common viruses in pigs with specific primers.

Virus	Primer Name	Sequence(5’-3’)
**PCV2** (DNA virus)	PCV2-F	GAATTGTACATACATGGTTA
	PCV2-R	CAAGGCTACCACAGTCAGAA
**PRV** (DNA virus)	PRV-F	GGTGGACCGGCTGCTGAACGA
	PRV-R	GCTGCTGGTAGAACGGCGTCA
**PRRSV** (RNA virus)	PRRSV-F	GCCAGTTCCAGCCAGTCAATCA
	PRRSV-R	GCCCCGATTGAATAGGTGAC
**PEDV** (RNA virus)	PEDV-F	GGTCTTTTTCGCTTTCAGCATCCT
	PEDV-R	CACTATCTGTGAGAACCGCACTCG

**Fig 6:**
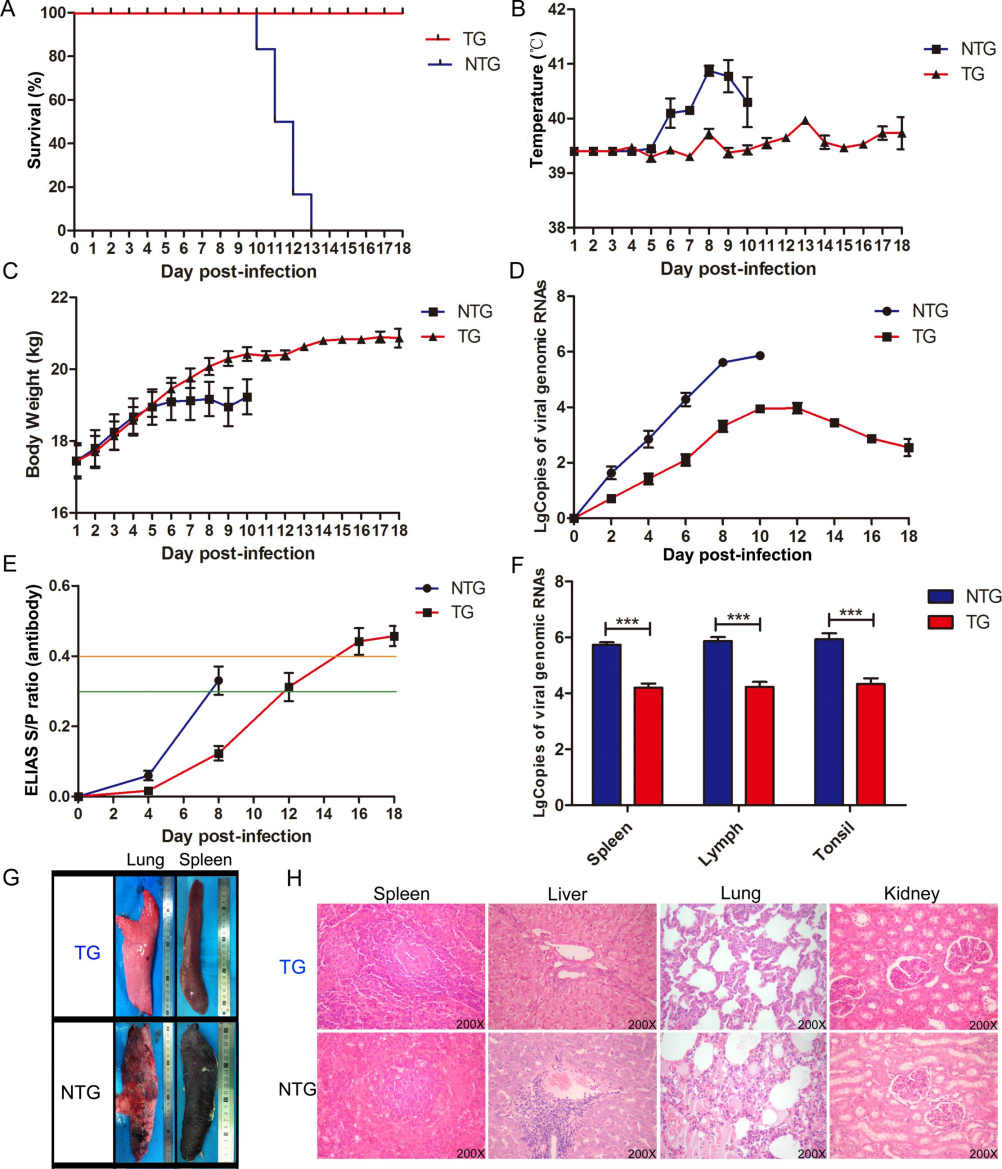
TG pigs exhibit antiviral responses during CSFV infection. (**A**) Survival curves of the challenged pigs during in-contact infection. Different curves indicate different pigs. The blue curve indicates the NTG pigs and the red curve indicates the TG pigs. The pigs in the TG group survived significantly longer than those in the NTG group. (**B**) The rectal temperatures of the pigs following challenge with CSFV (TG group, n = 6; NTG group, n = 6). The rectal temperatures were measured daily until the animals died. Error bars represent the standard deviations of at least 3 biological replicates. (**C**) Body weight curves for the challenged pigs during in-contact infection. Error bars represent the standard deviations of at least 3 biological replicates. (**D**) Viral replication curves in the challenged pigs. CSFV genome copies in the blood samples are presented according to the standard curve. Data in the panels are presented as the mean ± SE. Error bars represent the standard deviations of at least 3 biological replicates. (**E**) Serum was collected from pigs on 0, 4, 8 and 16 dpi to analyse antibodies against CSFV using a commercial CSFV Antibody Test Kit (IDEXX). ELISA plates were read using a 450nm filter on an ELISA reader to determine optical density, and these values were used to calculate the percent inhibition (PI). Error bars represent the standard deviations of at least 3 biological replicates. A sample was considered positive for CSFV antibody when the PI was ≥ 40% (orange line), potentially positive when 30% < PI< 40% and negative when the PI was optical density ≤ 30% (green line). (**F**) Viral RNA copies present in major susceptible tissues from the challenged animals (NTG-1 and TG-4) were evaluated by real-time PCR. Viral RNA expression was analysed with an unpaired t-test (***p<0.001). Error bars represent the SEMs, n = 3. (**G**) Histopathological changes in different tissues and organs between the NTG and TG pigs are indicated in the photographos. (**H**) Histopathological changes in the NTG pigs were confirmed by HE staining. These histopathological changes included a decrease in splenic white pulp and hyperaemia; an expansion of splenic red pulp; acidophilic change and accumulation of lipid droplets in hepatocytes; infiltration of inflammatory cells in the portal area of the liver; alveolar effusion, bleeding and infiltration of a large number of inflammatory cells in the lungs; and unclear renal tubular epithelial cell boundaries; and cell cavitation in the kidneys.

During viral challenge, as early as 1 day post-infection (dpi), we found that all of the injected NTG-In pigs developed inappetence, lethargy, and severe diarrhoea and fever symptoms. At 7 dpi, the two injected NTG-In pigs died directly due to infection, and shortly after, all of the NTG pigs died between 10 and 13 dpi. In contrast, all of the TG pigs remained alive until they were euthanasia (Fig 6A). Next, we investigated the body temperatures and daily body weights of these challenged pigs. These NTG pigs exhibited more severe fever symptoms (Fig 6B) and slower weight gain (Fig 6C) than the TG pigs. Furthermore, blood samples were collected every 2 days to analyse the dynamic characteristics of the virus in these challenged pigs. Notably, the levels of viral RNA were higher in the NTG pigs than in the TG pigs (Fig 6D), suggesting that the TG pigs could effectively alter the dynamic characteristics of the viral infection. The antibody response in the challenged pigs was further measured every 4 days until day 16 (Fig 6E). The results showed that the antibody response in the NTG pigs was higher than that in the TG group, and compared to the NTG pigs, the antibody response in the TG pigs was delayed by 4~5 days. Meanwhile, compared to the NTG pigs, we found that viral RNA maintained low levels of expression in CSF susceptible tissues from the TG pigs (Fig 6F). Additionally, we found that the average time to initial CSFV-associated clinical manifestation for the NTG and TG pigs were 4 and 8.5 dpi, respectively (S9C Fig). Furthermore, when the last two NTG pigs (NTG-1 and NTG-3) developed severe symptoms (anorexia, conjunctivitis, severe diarrhoea, fever, convulsions and heamorrhage) and were humanely euthanized at 13 dpi, major tissues were collected for histopathological analysis. Finally, the histopathological changes in these tissues (Fig 6G, S9D Fig) were confirmed by haematoxylin/eosin (HE) staining (Fig 6H). Together, these results indicated that there were significant differences in typical signs of CSFV infection and mortality between the TG and NTG pigs during viral challenge, suggesting that the replication of CSFV could be effectively inhibited in the TG pigs. Next, we evaluated the potential viral escape events in the challenged pigs using blood samples (the blood samples were isolated from four euthanized TG pigs at 18 dpi). Sequencing results indicated that there was no mutation or a partial or complete deletion within the target sequences and the flanking regions (S10 Fig).

## Discussion

CSF is one of the most economically important infectious diseases affecting pigs worldwide. Due to the economic importance of this virus, intensive control strategies for CSFV have been implemented for several decades, but the disease is still listed by OIE (OIE 2017). There are several possible reasons for the failure to stamp out the disease: (a) the virulence of CSFV is a complex and multifactorial phenomenon that has not been completely characterized; (b) the acquired strategies of viral evasion of the host antiviral response require further in-depth research; (c) the impacts of geography, climate, national policy and people’s awareness of the elimination of disease must be considered; (d) there are limitations associated with current commercial vaccines; (e) the control of wild boar reservoirs is a significant challenge. Additionally, the singleness of control strategies based on vaccination may also be a contributing factor. Therefore, there remains a long way to before elimination of the virus. As an alternative, the breeding of anti-CSFV pigs via a genome editing-based strategy could be a direct and effective approach, which would facilitate the permanent introduction of novel disease resistance traits into the mass population of production pigs via conventional breeding techniques.

Random integration-based TG technology could be used for the production of TG pigs, however the copy number cannot be regulated by this method, and random integration often leads to unpredictable gene expression and unstable phenotypes. Although traditional homologous recombination (HR) can be used to generate TG pigs, this technique is time consuming, inefficient and laborious. Recently developed programmable genome editing (PGE) technologies, such as TALEN and CRISPR/Cas9, have been widely used to produce TG animals. Many gene editing strategies can be used to produce viral disease-resistant pigs, and these approaches include knock-out or replacement of attachment factors or receptors (heparan sulfate, sialoadhesin, CD163, etc.) involved in viral infection [45–47], and the inhibition of viral replication via the overexpression of antiviral genes (RSAD2, etc.)[48–50]. Compared with other antiviral strategies, RNAi technology has some innate advantages. For example, regardless of other effects (e.g., the modification of viral receptors may affect normal physiological and biochemical functions, and these modified receptors may be vulnerable to invasion by other diseases), RNAi technology is a simple in design and exhibits target specificity and flexibility to target one or more loci that are completely conserved and essential for the replication and proliferation of virus and their serotypes [51]. However, the most challenging trouble in RNAi application is the delivery system, and the studies on RNAi-mediated anti-CSFV activity have been conducted mainly in vitro. Furthermore, whether the knock-in of shRNA at *pRosa26* locus in pigs can confer permanent resistance against CSFV infection remains unclear [52–54].

In this study, we successfully produced anti-CSFV pigs via CRISPR/Cas9-based knock-in technology. Our study showed that the shRNA site-specific insertion at the *pRosa26* locus can be consistently driven by the endogenous *pRosa26* promoter. The in vitro and in vivo viral challenge experiments demonstrated that these TG pigs could exhibit greater resistance to CSFV infection than NTG pigs, and the acquired RNAi-based antiviral ability in these TG founders could be stably transmitted to their F1-generation offspring. However, even though the extent and severity of clinical signs and viremia were lower in the TG pigs than in the NTG pigs, some clinical signs and viremia were also observed in the TG pigs. This findings suggests that the antiviral activity was limited to the single-copy shRNA gene, and multiple antiviral shRNAs strategy may be better at permanently blocking virus replication and preventing the emergence of resistant variants[42,43]. On the other hand, some host factors (LamR[55], PCBP1, HB[56], HSP70[57], etc.) that are essential for viral replication can also be targeted, and this strategy may be used to further reduce the chance of viral escape. Furthermore, to achieve improved antiviral effects, different antiviral strategies should be combined.

The off-target effect is a major concern with the Cas9-mediated gene editing technology, due to CRISPR/Cas9 can tolerate small numbers of mismatches between sgRNA and the target region, particularly when the mismatch is 8–12 bases away from the protospacer adjacent motif (PAM)[58]. Our results indicated that CRISPR/Cas9 does not induce detectable off-target mutation in our study. Nevertheless, off-target mutations may occur at sites beyond those predicted loci, hence, a comprehensive analysis, such as whole-genome sequencing, would be an essential component of future efforts to establish the safety of this approach. Considering that the off-target effect is site-dependent and can be predicted and likely minimized by general design guidelines[59], and more specific strategies using improved Cas9 have already been established [60–62], suggesting CRISPR/Cas9-based genome editing technology will be a reliable strategy for genetically modified pigs.

Recently, some mammalian viral proteins, such as IAV[63], NS1[64] and Ebola virus (EBOV) VP35[65], have been reported to suppress RNAi in vitro, preventing the elimination of viral RNAs [63,66,67]. However, it remains unclear whether CSFV can encode the suppressors of RNAi (VSRs), and whether the VSRs could suppress the attained antiviral RNAi character in the TG pigs. To address these important questions, further in-depth and systematic research will be performed in subsequent studies, and the antiviral strategy present in the TG pigs may be used to develop an infection model for RNAi suppression.

Further development of transgenic disease resistance in farm animals will undoubtedly stimulate debate about the application of this technology in food production. Technically, in this study, we demonstrated the generation of TG pigs based on CRISPR/Cas9-mediated homology directed repair (HDR). The targeting vector used in this study was promoter-less, which could prevent further problems, such as unstable phenotypes, unpredictable gene expression and oncogene activations, and the antiviral shRNA gene in TG pigs was driven by the endogenous *pRosa26* promoter, which drives exogenous gene expression in a consistent and stable manner by preventing DNA methylation. Additionally, to reduce the potential risk associated with drug selection and to increase the biological safety of the TG pigs, no selectable maker genes were introduced during the generation of the TG pigs. All the above factors suggest that the antiviral strategy can help provide market support.

Moreover, we believe that TG pigs have substantial potential advantages over vaccination. The immunization of pigs confers effective protection against CSFV, and the induction of complete clinical protection takes at least 7 days, during which, the body may become so overrun with infection that the immune system of the pig may stop resisting the infection. In this study, we observed that the time at which the CSFV-associated clinical symptoms began to appear in the TG pigs was significantly delayed (4~5 days) compared with that in the NTG pigs. These findings suggest that these TG pigs may have more time than NTG pigs to evoke protective immunity and combat the virus. Overall, the integrated strategy may be preferable over the singleness of control strategies based on vaccination, and should be considered. Additionally, the CSFV genome is a positive-sense, single-stranded RNA that functions as both messenger RNA (mRNA) and a replication template. These TG pig and TG strategy could be useful resources for scientists and helping them better understand and study RNAi. Nevertheless, admittedly, the animal challenge experiment was performed on a small-scale and this was a short-term preliminary study. Additionally, it is important to assess any genetic modification for potential hazards. We are conducting long-term studies to monitor the antiviral ability and gene editing on these animals as they age and mature.

In summary, in this proof-of-principle study, we demonstrated the combinatorial application of CRISPR/Cas9 technology and RNAi to generate TG pigs. Viral challenge experiments confirmed that these TG pigs could effectively limit the replication of CSFV in vivo and in vitro and that the disease resistance traits in the TG founders could be stably transmitted to their F1-generation offspring. We believe that the use of TG pigs can contribute to reduction of CSFV-related economic losses and could have financial benefits. Additionally, this antiviral strategy is technically applicable to other domestic species and will provide insights for future antiviral research.

## Materials and methods

### Ethics statement

All animal studies were approved by the Animal Welfare and Research Ethics Committee at Jilin University (Approval ID: 20160602), and all procedures were conducted strictly in accordance with the Guide for the Care and Use of Laboratory Animals. All surgeries were performed under anesthesia, and every effort was made to minimize animal suffering.

### Cells and virus

Porcine kidney cell line-15 (PK-15) cells (Lot Number: 58808810 ATCC Number: CCL-33) were cultured in Dulbecco’s modified Eagle’s medium (DMEM) supplemented with 5% fetal bovine serum (FBS) (Gibco, Grand Island, New York, USA) and incubated at 39°C in an atmosphere of 5% CO2. PFF cells were cultured in DMEM supplemented with 10% FBS and incubated at 39°C in an atmosphere of 5% CO2. PK-15 cells were not included in the list of commonly misidentified cell lines maintained by the International Cell Line Authentication Committee. The origin of the cells (sus scrofa, epithelial) was confirmed by PCR in RIKEN BRC (link of datasheet: http://www2.brc.riken.jp/lab/cell/detail.cgi?cell_no=RCB0534&type=1). The cells were negative for mycoplasma by both PCR and nuclear staining, which were performed based on protocols by RIKEN BRC (http://cell.brc.riken.jp/ja/quality/myco_kensa). CSFV (strain Shimen) and the positive anti-CSFV serum were kindly provided by Dr. Changchun Tu (Academy of Military Medical Sciences, Changchun, China).

### Selection of siRNAs and IFA

To reduce the chance of viral escape, all the designed and synthesized siRNA-target sequences are essential and well-conserved among different CSFV strains [68,69]. All siRNAs were designed and synthesized by Suzhou Genema (Suzhou, China). Then, these siRNAs were individually introduced into PK-15 cells by electroporation, at a siRNA final concentration of 200 nM. Five hours post-transfection, the siRNA-transfected PK-15 cells were inoculated with CSFV and cultured in DMEM with 5% (v/v) fetal bovine serum (FBS) at 39°C and 5% CO2. 72 hours later, the proliferation of CSFV in siRNA-transfected PK-15 cells was determined by IFA. Briefly, siRNA-transfected PK-15 cells seeded in 24-well plates with four replicates for each siRNA. At 70–80% confluency, the cells were infected with CSFV (200 TCID_50_ per well). At 2 h post-inoculation (hpi), the medium was removed and the cells were cultured in fresh DMEM supplemented with 3% fetal bovine serum. 72 hours later, PK-15 cells were washed three times with cold phosphate-buffered saline (PBS). Then, the cells were fixed in 80% (v/v) cold acetone for at least 30 min in -20°C/-80°C refrigerator. Next, the fixed cells were washed five times by phosphate-buffered saline with Tween 20 (PBST) and incubated with anti-E2 polyclonal antibody (PAb) (1:100) for 2 h at 37°C, washed five times with PBS, and incubated with a fluorescein isothiocyanate (FITC)-labeled goat anti-pig IgG (1:100) antibody (catalog no. F1638; Sigma-Aldrich) for 30 min at 37°C. After five washes with PBS, the cells were examined using a fluorescence microscope Eclipse TE2000-V (Nikon Imaging, Japan).

### Plasmids

sgRNAs that targeted the p*Rosa26* locus were designed using online software, and sgRNA oligonucleotides were annealed and cloned into the PX330 vector (42230, Addgene) using the method described by Zhang at the Broad Institute of MIT. Targeting sgRNAs were designed and synthesized by Comate Bioscience Co.,Ltd. (Changchun, China). Two complementary sgRNA oligo DNAs were synthesized and then annealed to double-stranded DNA in the presence of 10 × NEB standard Taqbuffer and this product was ligated into the BbsI sites of the vector backbone to form the intact targeting plasmid.

The targeting vector contained a 0.5 kb left homology arm (HA) and a 1.0 kb right HA (S3A Fig). The HAs were amplified by genomic PCR and cloned into the PUC57 vector. The shRNA gene was subsequently inserted between the right and left arms.

### Isolation and culture of PFFs

Twelve 33-day-old fetuses were separated from Large White sows in the gestation period, and primary PFFs were isolated from these 33-day-old foetuses of Large White pigs. After removal of the head, tail, limb bones and viscera from the foetal body, the fetuses were cut into small pieces, digested with a sterile collagenase solution and cultured in DMEM supplemented with 20% FBS at 39°C and 5% CO2 in a humidified incubator.

### Electroporation of PFFs and selection of PFF cell clones

Approximately 3 × 10^6^ PFFs and the corresponding plasmids (30 μg of targeting vector, 30 μg of PX330 vector) were suspended in 300 μL of Opti-MEM (Gibco, Grand Island, New York, USA) in 2 mm gap cuvettes, and electroporated by using specified parameters with a BTX-ECM 2001. The cells were inoculated into ten 100 mm dishes at 48 h post-transfection, and the cell inoculation density per 100 mm dishes was 3,500 cells/dish on average. The cell clones were picked and cultured into 24-well plates. After a confluence of 80% or more was reached, 15% of each cell clone was digested and lysed with 10 μl of NP40 lysis buffer (0.45% NP40plus 0.6% proteinase K) for 1 h at 56°C and 10 min at 95°C. The lysate was used as the PCR template and was subjected to 1% agarose gel electrophoresis. Additionally, the knock-in events were confirmed by PCR with specific primers (Table 4). The positive cell clones were thawed and cultured in 12-well plates before SCNT.

**Table 4:** Knock-in primers and corresponding sequences.

Primer Name	Sequence Name	Sequence(5’-3’)
KI-1	F3	GAAGGGAGATAGGTTAAAAAGC
	R3	ATTCAAAAGACATAAAGGGGAG
KI-2	F2	GGTCCCAAATGAGCGAAAC
	R2	AGCGAGCACTTAACAAGGC
KI-3	F7	GATACATTTTTACAAAGCTGAATTA
	R7	CACTACCAAACATACAAAAGAACTA

### SCNT

The shRNA knock-in positive PFF cells were selected with the limiting dilution method. The positive cells were used for somatic cell nuclear transfer as described previously [38]. Reconstructed embryos were then surgically transferred into the oviducts of surrogate females on the first day of standing estrus. The pregnancy status was monitored using an ultrasound scanner between 30–35 days post-transplantation. Some embryos were cultured for 6–7 days to test the blastocyst formation rate and developmental ability.

### Generation of TG pigs and Southern blotting analyses

shRNA knock-in colonies derived from individual cells were obtained with the limiting dilution method (Xie et al., 2017). These positive cell clones were used as nuclear donor cells to generate transgenic pigs by SCNT. Approximately 250 embryos were transferred into each surrogate pig, and transgenic pigs were delivered by natural birth at full term. Transgene integration was identified by PCR analysis with specific primers. To confirm transgenic insertion into the pig genome, Southern blot was performed by Southern Blot Services (ZooNBIO Biotechnology). DNA was isolated from the TG piglet and WT pig tissues and digested with BamHI. shRNA targeting vector was used as a positive control. The probe was hybridized to a 20.424-kb fragment, which is depicted in Fig 2B, indicating site-specific gene insertion.

### siRNA expression level analysis

Small RNAs were isolated by using the miRcute miRNA Isolation Kit (Tiangen, Beijing, China). From purified RNA, complementary DNA was synthesized using the miRcute miRNA First-Strand cDNA Synthesis Kit (Tiangen, Beijing, China). RT-PCR was performed with specific primers. Quantitative RT-PCR was also performed using the miRcute miRNA qPCR Detection Kit (Tiangen, Beijing, China) according the manufacturer′s instructions. SYBR Green real-time PCR was performed using the BIO-RAD IQ5 multicolor real-time PCR detection system. shRNA expression was normalized to the expression of endogenous U6 using the 2−ΔΔCt method.

### Molecular beacon assay

shRNA-specific MB design [70], the MB loop sequence (GGCTAATCCACTTCAGGGTTC) is complementary to the targeting siRNA, and the MB stem sequence (CCTCC) is typically five nucleotides. Then, an appropriate dye-quencher pair is selected (CY 3 fluorophore & Blank Hole quencher 2), and conjugate the dye and quencher to the 5′and 3′ends of the MB sequence, respectively. Prepare total RNA from TG cells and NTG cells, and normalize the total RNA to GAPDH. Then, establish a dose-dependence curve using the serial dilutions of MBs and select optimal concentration for further testing. The MB signal at the highest target oligonucleotide concentration should generally be 5–30 times higher than the background signal quantified in the negative control experiment in which the signal level of MBs without any target is measured. Add 50 μl of MB solution to each well of a 96-well black-bottomed plate, and then add 50 μl solutions containing the target oligonucleotide to their designated wells. Incubate at 37°C for 5 min to allow the solutions to equilibrate. The fluorescence intensity of MBs is detected by using a microplate reader.

### Determination of transgene copy number

The copy number of antiviral shRNA gene was determined by qPCR as previously described [71]. Briefly, a standard curve was produced with series of standard samples containing 0, 1, 2, 4, 8, 10 copies of the shRNA gene, respectively, by mixing the wild-type genome of pig with shRNA expression vector. The absolute quantitative standard curve was drawn by plotting ÄCt = Ct_shRNA_−Ct_TFRC_ against the log of shRNA gene copies of corresponding standard samples.

### Viral challenge assay in TG cells

The in vitro viral challenge assay was strictly performed at a designated safe place. TG fibroblasts, TG kidney cells and TG umbilical vein endothelial cells were isolated from newborn TG pigs. These cells, cultured in 24-well plates, were inoculated with 200 TCID_50_ of CSFV (Shimen strain), and there were four replicates for each TG cell types. One hour later, the inoculums were replaced with fresh medium (5% fetal bovine serum). After 48-h incubation, cells and virus were collected and evaluated by IFA and qPCR. To analyze CSFV proliferation in TG cells by qPCR, total RNA was extracted from the CSFV-infected cells using TRIzol-A+ reagent (Tiangen, Beijing, China) and reverse transcribed into cDNA using the BioRT cDNA First Strand Synthesis Kit (Bioer, Hangzhou, China) according to the manufacturer’s protocol. SYBR Green real-time PCR was performed using the BIO-RAD IQ5 multicolor real-time PCR detection system and the BioEasy SYBR Green I real-time PCR kit.

### Viral challenge assay in TG pigs

All animal studies were performed according to protocols approved by the animal Welfare Committee of China Agricultural University. All pigs (the NTG-In group (n = 2), NTG group (n = 6) and TG group (n = 6)) were 55 days old and separated into two rooms. The pigs in the NTG and TG groups were same age pigs from the F0 generation of the TG founders. Before the CSFV challenge, all pigs were confirmed to be CSFV negative, and a commercial CSFV enzyme-linked immunosorbent assay kit (ELISA; IDEXX Laboratories, Inc., Westbrook, ME, USA) was used to test CSFV antibodies in these pigs. The NTG-In pigs were challenged by intramuscular injection in the neck with 1.0×10^4^ TCID_50_ CSFV Shimen in 2.5 ml of PBS. The in vivo viral challenge assay was strictly performed at a designated safe place. Then, the transgenic animal corpses were received humane treatment when the experiments were completed.

### Quantification of serum viral RNA

Quantitative RT-PCR was performed to examine CSFV in pig blood. Blood samples from each pig were collected at days 0, 2, 4, 6, 8, 10, 12, 14, 16 and 18 after injection. Viral genomic RNA was isolated by using Trizol (Tiangen, Beijing, China) according to the manufacturer’s instructions. A standard curve was generated to detect the viral load in each blood sample with 10-fold serial dilutions of viral lysates ranging from 10^8^ to 10^2^. SYBR Green real-time PCR was performed using the BIO-RAD IQ5 multicolor real-time PCR detection system and the BioEasy SYBR Green I real-time PCR kit and the Ct values and CSFV RNA copies were determined.

### Histopathological analysis

All animals were killed on the 10th day post-infection. Major tissues, including the heart, spleen, lung and other tissues, from the pigs were fixed in formalin followed by routine paraffin sectioning and HE staining. Histopathological changes were observed under a microscope.

## Supporting information

S1 Fig Main experimental strategies for the generation of antiviral TG pigs. (A) Designing and selecting shRNAs that could efficiently inhibit CSFV. (B) Construction of CRISPR/Cas9 expression plasmid and shRNA targeting donor plasmid. (C) The PFFs was transfected with these plasmids by electroporation. (D) Selecting transgenic cell clones via limiting dilution method. (E) The antiviral activities of these transgenic PFFs were examined by viral challenge assay. (F) These transgenic PFFs were further used to generate transgenic pigs via SCNT. (G) The reconstructed embryos were transferred into surrogate sows via embryo transfer. (H) Production of F0-generation transgenic pigs. (I) Production of F1-generation transgenic pigs. (J) Animal challenge experiments were performed in these transgenic pigs.
(TIF)

S2 Fig Selection of the targeting siRNA. (A)The antiviral activity of various designed siRNAs (siRNA-C1~siRNA-C10) was assessed by IFA in siRNA-transfected PK-15 cells at 72 h post-infection. The cells cultured in 24-well plates were inoculated with 1000 TCID_50_ of CSFV (Shimen strain). At 72 hpi, the CSFV-infected cells were incubated with an E2-specific antibody (PAb) and then stained with a fluorescein isothiocyanate (FITC)-labelled goat anti-pig IgG (1:100). Cells were analyzed under fluorescence microscope. siRNA-Scr: scrambled siRNA. NC: negative control (no CSFV). (B) Scheme depicting site-specific shRNA targets in the CSFV genome and the target sequences of si-C3 and si-C6.
(TIF)

S3 Fig Verification of site-specific knock-in events in PFF cell clones. (A) Composition and structure of the targeting vector for knock-in. 5’HA: left homologous arm; 3’HA: right homologous arm; shRNA: anti-CSFV shRNA gene cassette. (B) Scheme for shRNA site-specific knock-in. HA: homology arm. (C) Sanger sequencing analyses were used to further confirm the EGFP site-specific knock-in events in the *pRosa26* locus.
(TIF)

S4 Fig Expression of the targeting siRNA and verification of antiviral ability in TG PK-15 cell clones. (A) Virus resistance in shRNA-C3 (#44) and shRNA-C6 (#65) transgenic PFFs was examined by IFA. At 72 hpi, the CSFV-infected cells were incubated with an E2-specific antibody (PAb) and then stained with fluorescein isothiocyanate (FITC)-labeled goat anti-pig IgG (1:100). Cells were analyzed under fluorescence microscope. shRNA-Scr: scrambled shRNA transgenic PFFs. WT: wild-type PFFs. (B) The replication and proliferation of CSFV in TG PK-15 cell clones were evaluated by IFA. Cells cultured in 24-well plates were inoculated with 200 TCID_50_ of CSFV (Shimen strain). At 72 hpi, the CSFV-infected cells were incubated with an E2-specific antibody (PAb) and then stained with fluorescein isothiocyanate (FITC)-labelled goat anti-pig IgG (1:100). Cells were analyzed under fluorescence microscope. shRNA-C3: shRNA-C3 knock-in PK-15 cells. shRNA-C6: shRNA-C6 knock-in PK-15 cells. shRNA-Scr, scrambled shRNA knock-in PK-15 cells. WT: wild-type PK-15 cells. (C) Sanger sequencing analyses were used to further confirm expression of the targeting siRNA in positive PFF cell clones. (D) CCK8 assay was used to evaluate the growth and proliferation of knock-in PFFs. (E) The expression levels of Some proinflammatory cytokines and interferons in TG PFF cells were measured by qRT-PCR. Error bars represent the SEMs, n = 3.
(TIF)

S5 Fig Phenotypic analyses of TG pigs. (A) Relative expression levels of the targeting siRNA (siRNA-C3) in various tissues and cells from TG pigs were detected by RT-PCR. (B) Three types of primary TG cells isolated from TG pigs. In particular, the isolated PUVECs were labelled with an anti-CD31 antibody and analysed by immunofluorescence.
(TIF)

S6 Fig Viral escape study in challenged TG cells. (A) The scheme for viral escape detection by PCR. Blue arrows indicate the primers used for PCR (B) Primer specificity were analyzed using PCR amplification and 1.5% agarose gel electrophoresis. The red arrow indicates the objective band (264bp). (C) Sanger sequencing analyses were used to detect the viral escape events in different TG cells.
(TIF)

S7 Fig Phenotypic analyses of F1 generation TG pigs. (A) The knock-in event of shRNA gene at the *posa26* locus in F1 generation TG pigs was confirmed by qPCR. Pigs 3900, 3902 and 3904 were F0-generation TG pigs, pigs 0042, 0049 and 0058 were F1-generation TG pigs, and pig 0044 was an NTG pigs. Data are the means of three replicates±SDs. (B) Karyotype analysis results indicated that these TG pigs had normal porcine diploid chromosome numbers (2n = 38). (C) Viral infection in isolated F1-generation primary TG cells was confirmed by RT-PCR. (D) Viral infection in isolated F1 generation primary TG cells was further confirmed by IFA. Cells cultured in 24-well plates were inoculated with 200 TCID50 of CSFV (Shimen strain). At 72 hpi, the CSFV-infected cells were incubated with an E2-specific antibody (PAb) and then stained with fluorescein isothiocyanate (FITC)-labelled goat anti-pig IgG (1:100). Cells were analyzed under fluorescence microscope.
(TIF)

S8 Fig Molecular beacon assay. (A) Schematic depiction of molecular beacons to detect the targeting siRNA in TG pigs. (B) The relative expression levels of the targeting siRNA in various tissues and cells in TG and NTG pigs were detected with molecular beacons. TG: transgenic pigs. NTG: wild-type pigs. Targeting siRNA expression was analysed with an unpaired t-test (**p<0.01; ***p<0.001). Error bars represent the SEMs, n = 3.
(TIF)

S9 Fig Results of the in vivo viral challenge assay. (A) Different mental states were observed among the challenged pigs. NTG indicates NTG pigs and TG indicates TG pigs. (B) Haemorrhagic signs were observed in different organs and tissues in NTG pigs. ① indicates skin, ② indicates lymph and ③ indicates spleen. (C) Statistical data regarding the time of initial morbidity among challenged pigs. n = 6. Graphs show the mean ± S.E.M. (D) Pathological changes were also observed in lymphoid tissue.
(TIF)

S10 Fig Viral escape study in challenged TG pigs. Sequencing results indicated that there was no mutation within the target sequences and flanking regions. NTG: challenged non-transgenic pigs; TG: challenged transgenic pigs.
(TIF)
