# Supplementary figures and images for: Genetically modified pigs are protected from classical swine fever virus
Source: PLoS Pathog. 2018 Dec 13;14(12):e1007193. doi: 10.1371/journal.ppat.1007193 (PMC6292579; doi:10.1371/journal.ppat.1007193)

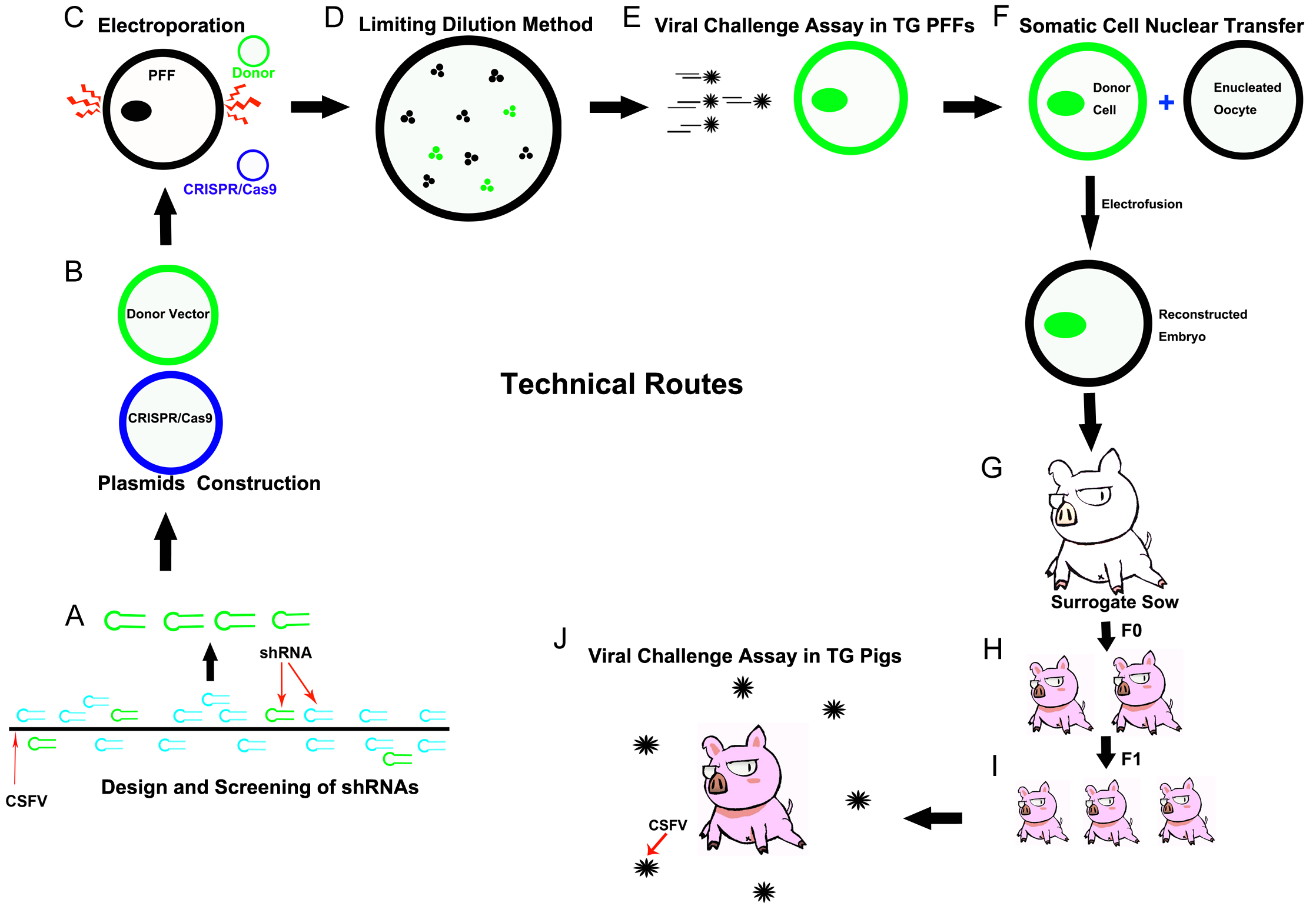

Supplement: S1 Fig — (A) Designing and selecting shRNAs that could efficiently inhibit CSFV. (B) Construction of CRISPR/Cas9 expression plasmid and shRNA targeting donor plasmid. (C) The PFFs was transfected with these plasmids by electroporation. (D) Selecting transgenic cell clones via limiting dilution method. (E) The antiviral activities of these transgenic PFFs were examined by viral challenge assay. (F) These transgenic PFFs were further used to generate transgenic pigs via SCNT. (G) The reconstructed embryos were transferred into surrogate sows via embryo transfer. (H) Production of F0-generation transgenic pigs. (I) Production of F1-generation transgenic pigs. (J) Animal challenge experiments were performed in these transgenic pigs. (TIF) [file ppat.1007193.s001.tif]

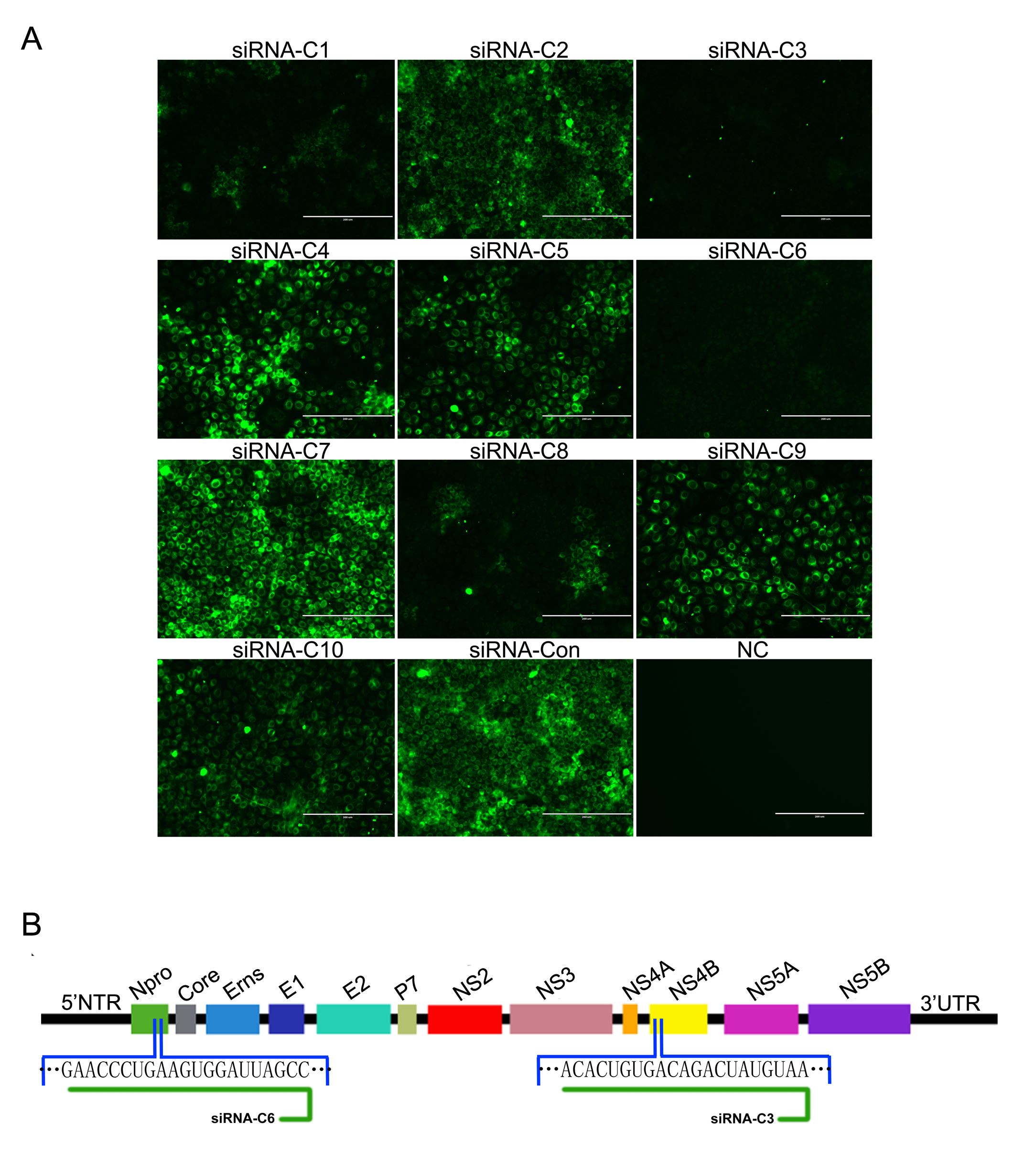

Supplement: S2 Fig — (A)The antiviral activity of various designed siRNAs (siRNA-C1~siRNA-C10) was assessed by IFA in siRNA-transfected PK-15 cells at 72 h post-infection. The cells cultured in 24-well plates were inoculated with 1000 TCID50 of CSFV (Shimen strain). At 72 hpi, the CSFV-infected cells were incubated with an E2-specific antibody (PAb) and then stained with a fluorescein isothiocyanate (FITC)-labelled goat anti-pig IgG (1:100). Cells were analyzed under fluorescence microscope. siRNA-Scr: scrambled siRNA. NC: negative control (no CSFV). (B) Scheme depicting site-specific shRNA targets in the CSFV genome and the target sequences of si-C3 and si-C6. (TIF) [file ppat.1007193.s002.tif]

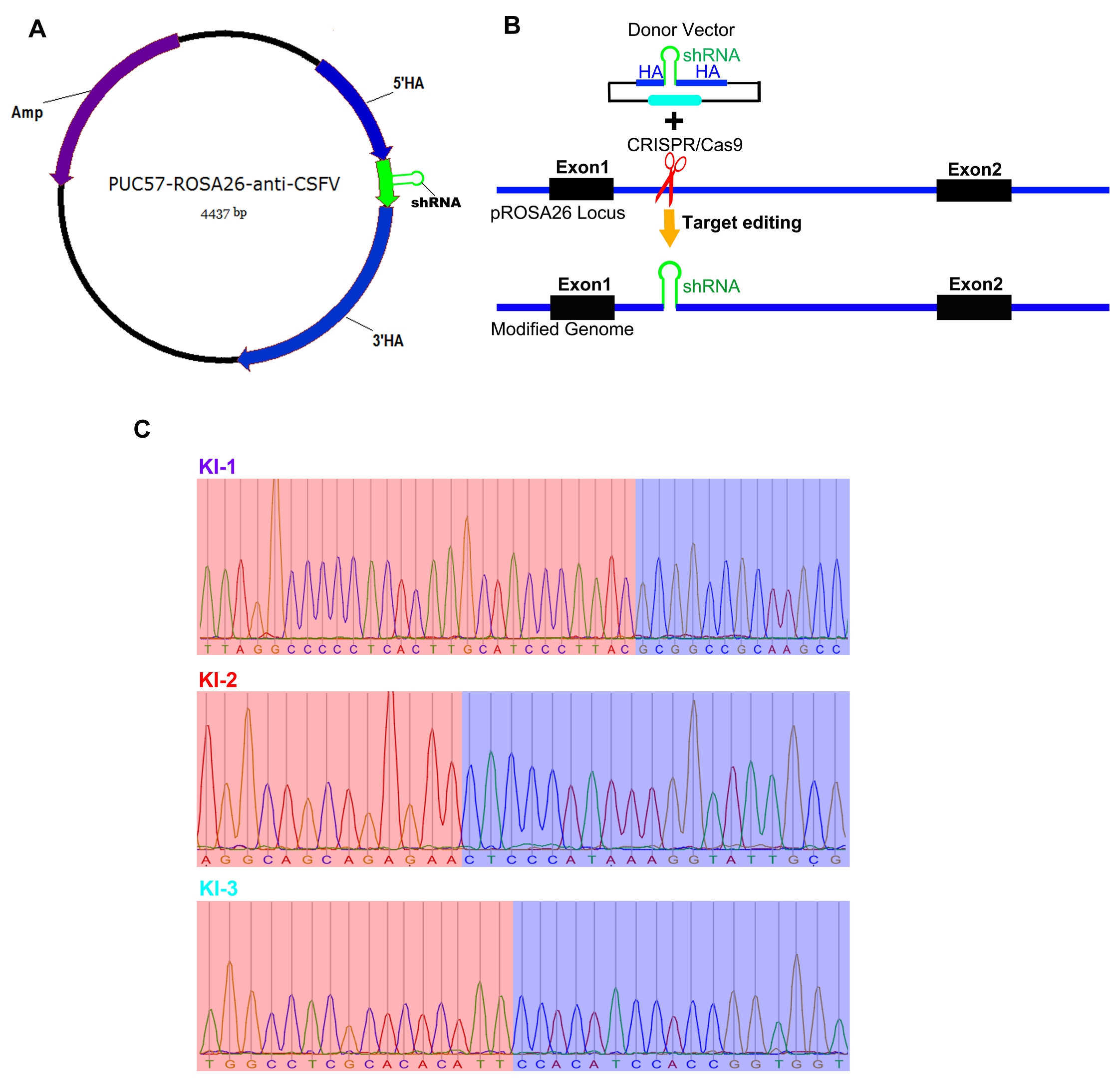

Supplement: S3 Fig — (A) Composition and structure of the targeting vector for knock-in. 5’HA: left homologous arm; 3’HA: right homologous arm; shRNA: anti-CSFV shRNA gene cassette. (B) Scheme for shRNA site-specific knock-in. HA: homology arm. (C) Sanger sequencing analyses were used to further confirm the EGFP site-specific knock-in events in the pRosa26 locus. (TIF) [file ppat.1007193.s003.tif]

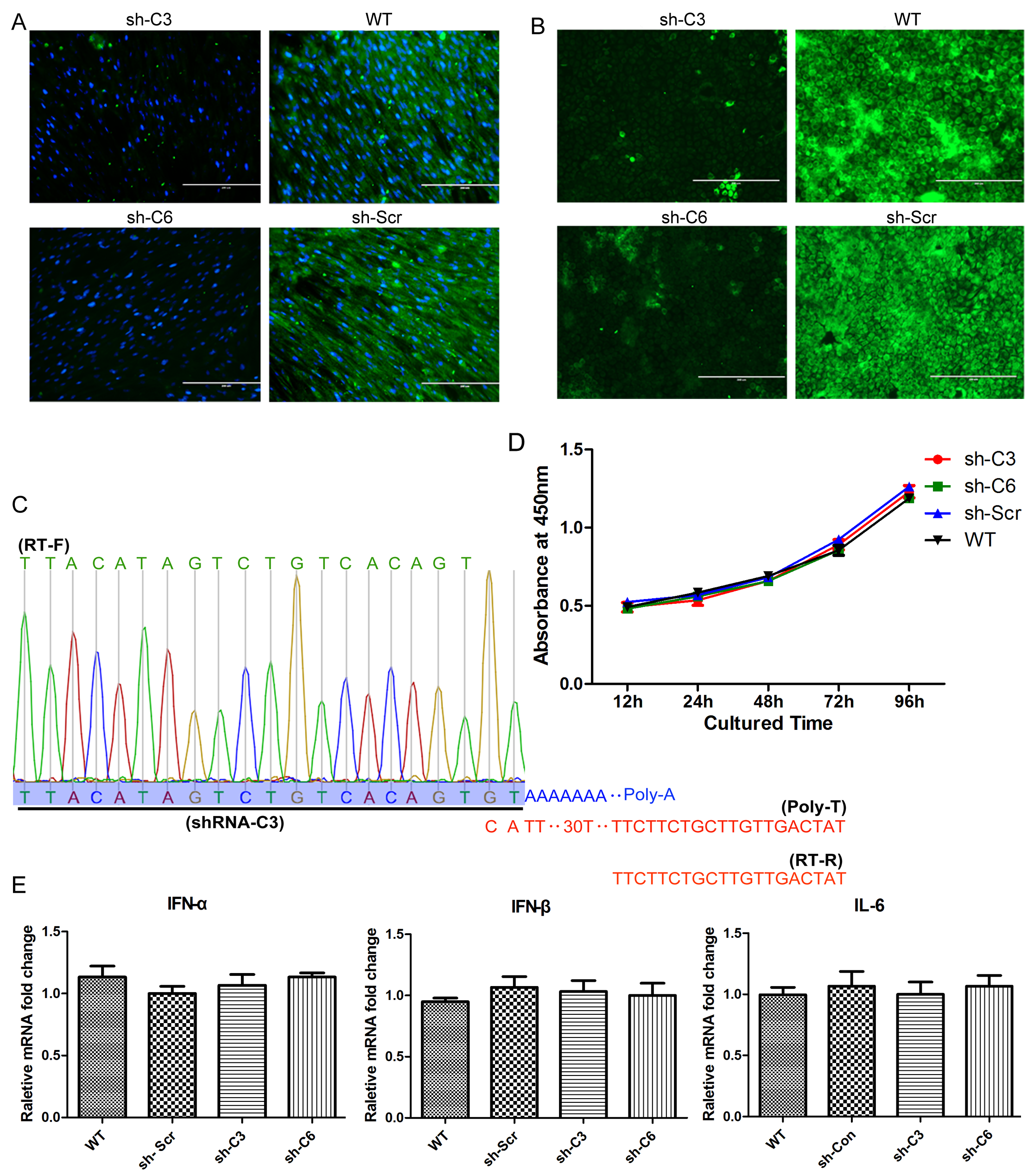

Supplement: S4 Fig — (A) Virus resistance in shRNA-C3 (#44) and shRNA-C6 (#65) transgenic PFFs was examined by IFA. At 72 hpi, the CSFV-infected cells were incubated with an E2-specific antibody (PAb) and then stained with fluorescein isothiocyanate (FITC)-labeled goat anti-pig IgG (1:100). Cells were analyzed under fluorescence microscope. shRNA-Scr: scrambled shRNA transgenic PFFs. WT: wild-type PFFs. (B) The replication and proliferation of CSFV in TG PK-15 cell clones were evaluated by IFA. Cells cultured in 24-well plates were inoculated with 200 TCID50 of CSFV (Shimen strain). At 72 hpi, the CSFV-infected cells were incubated with an E2-specific antibody (PAb) and then stained with fluorescein isothiocyanate (FITC)-labelled goat anti-pig IgG (1:100). Cells were analyzed under fluorescence microscope. shRNA-C3: shRNA-C3 knock-in PK-15 cells. shRNA-C6: shRNA-C6 knock-in PK-15 cells. shRNA-Scr, scrambled shRNA knock-in PK-15 cells. WT: wild-type PK-15 cells. (C) Sanger sequencing analyses were used to further confirm expression of the targeting siRNA in positive PFF cell clones. (D) CCK8 assay was used to evaluate the growth and proliferation of knock-in PFFs. (E) The expression levels of Some proinflammatory cytokines and interferons in TG PFF cells were measured by qRT-PCR. Error bars represent the SEMs, n = 3. (TIF) [file ppat.1007193.s004.tif]

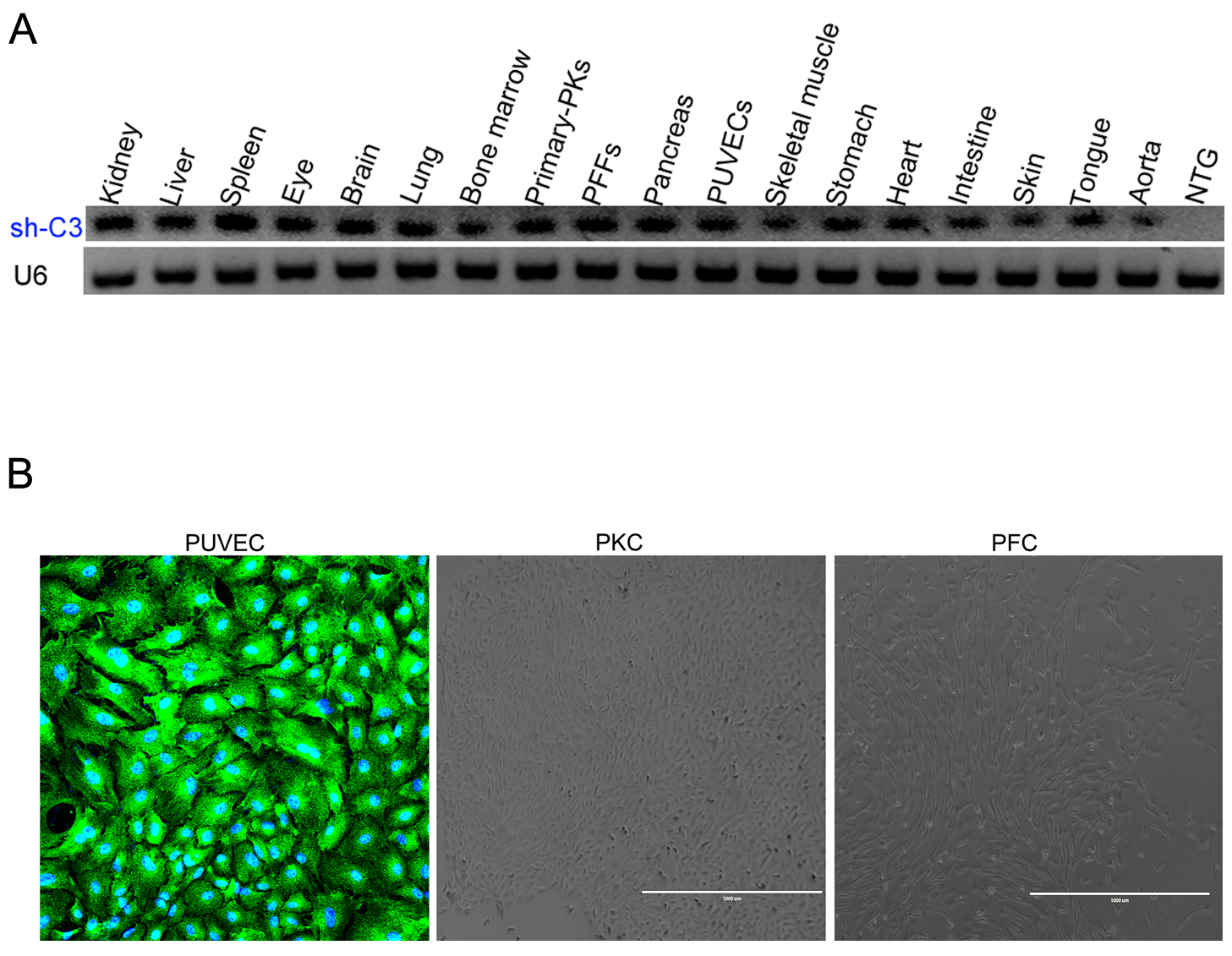

Supplement: S5 Fig — (A) Relative expression levels of the targeting siRNA (siRNA-C3) in various tissues and cells from TG pigs were detected by RT-PCR. (B) Three types of primary TG cells isolated from TG pigs. In particular, the isolated PUVECs were labelled with an anti-CD31 antibody and analysed by immunofluorescence. (TIF) [file ppat.1007193.s005.tif]

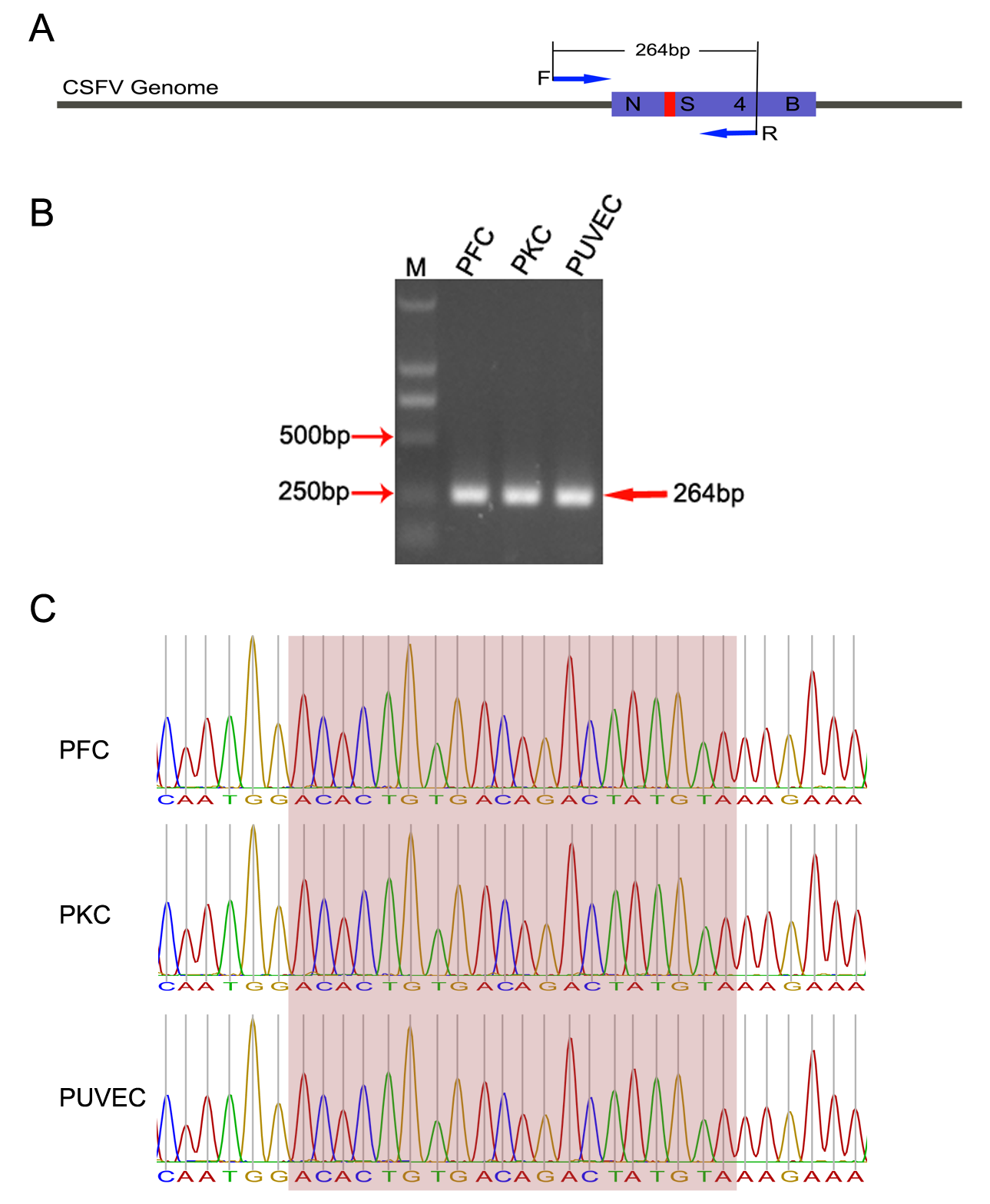

Supplement: S6 Fig — (A) The scheme for viral escape detection by PCR. Blue arrows indicate the primers used for PCR (B) Primer specificity were analyzed using PCR amplification and 1.5% agarose gel electrophoresis. The red arrow indicates the objective band (264bp). (C) Sanger sequencing analyses were used to detect the viral escape events in different TG cells. (TIF) [file ppat.1007193.s006.tif]

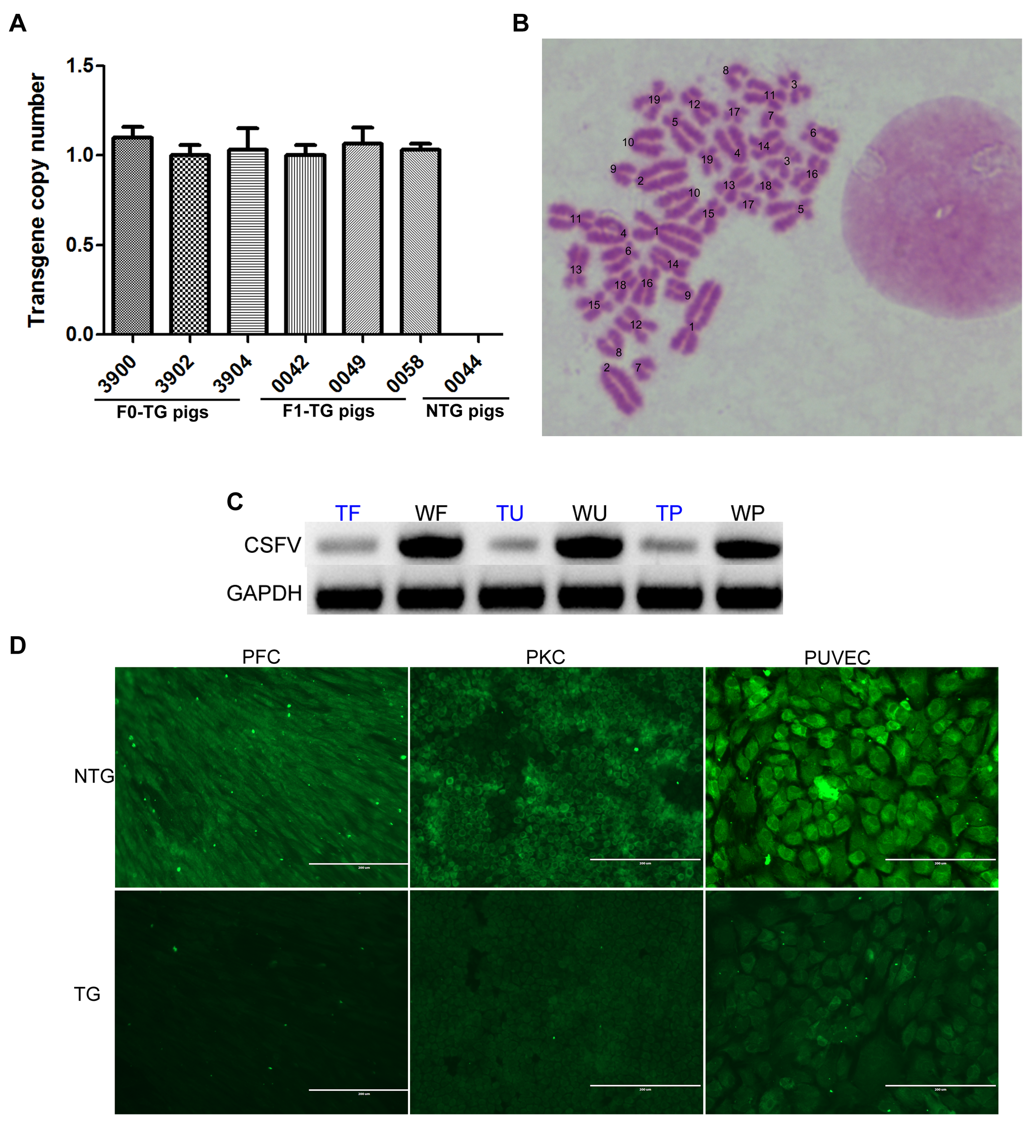

Supplement: S7 Fig — (A) The knock-in event of shRNA gene at the posa26 locus in F1 generation TG pigs was confirmed by qPCR. Pigs 3900, 3902 and 3904 were F0-generation TG pigs, pigs 0042, 0049 and 0058 were F1-generation TG pigs, and pig 0044 was an NTG pigs. Data are the means of three replicates±SDs. (B) Karyotype analysis results indicated that these TG pigs had normal porcine diploid chromosome numbers (2n = 38). (C) Viral infection in isolated F1-generation primary TG cells was confirmed by RT-PCR. (D) Viral infection in isolated F1 generation primary TG cells was further confirmed by IFA. Cells cultured in 24-well plates were inoculated with 200 TCID50 of CSFV (Shimen strain). At 72 hpi, the CSFV-infected cells were incubated with an E2-specific antibody (PAb) and then stained with fluorescein isothiocyanate (FITC)-labelled goat anti-pig IgG (1:100). Cells were analyzed under fluorescence microscope. (TIF) [file ppat.1007193.s007.tif]

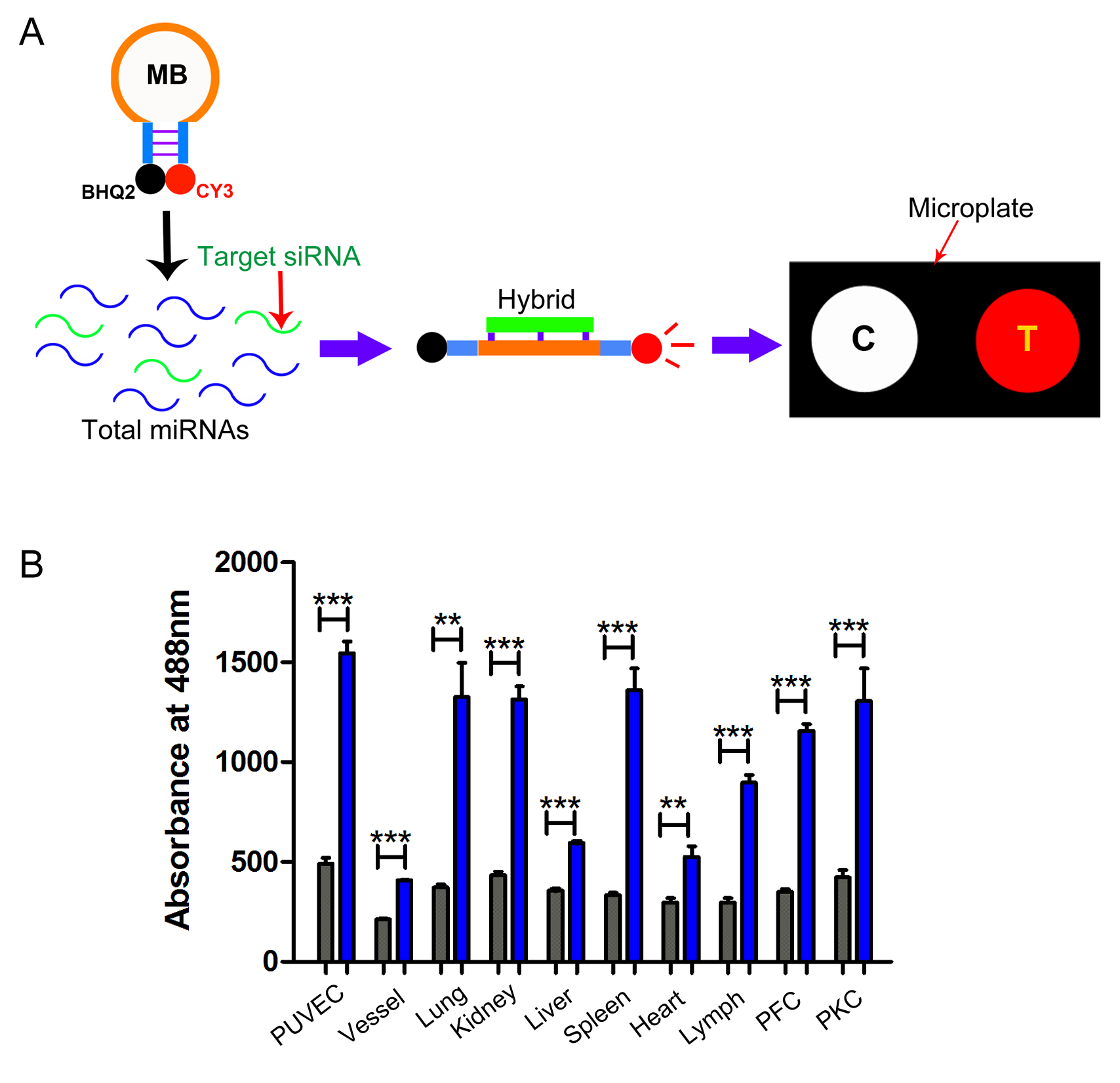

Supplement: S8 Fig — (A) Schematic depiction of molecular beacons to detect the targeting siRNA in TG pigs. (B) The relative expression levels of the targeting siRNA in various tissues and cells in TG and NTG pigs were detected with molecular beacons. TG: transgenic pigs. NTG: wild-type pigs. Targeting siRNA expression was analysed with an unpaired t-test (**p<0.01; ***p<0.001). Error bars represent the SEMs, n = 3. (TIF) [file ppat.1007193.s008.tif]

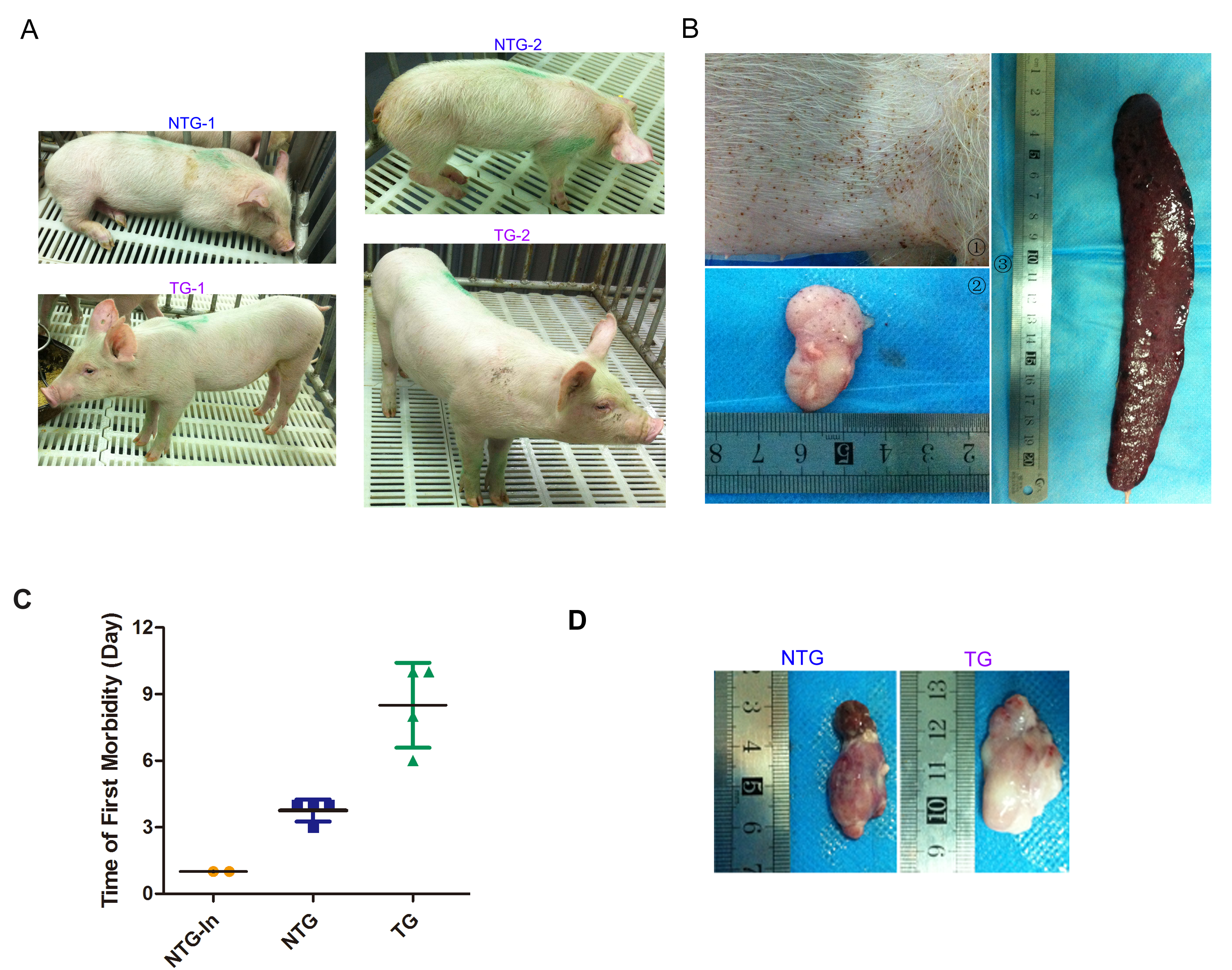

Supplement: S9 Fig — (A) Different mental states were observed among the challenged pigs. NTG indicates NTG pigs and TG indicates TG pigs. (B) Haemorrhagic signs were observed in different organs and tissues in NTG pigs. ① indicates skin, ② indicates lymph and ③ indicates spleen. (C) Statistical data regarding the time of initial morbidity among challenged pigs. n = 6. Graphs show the mean ± S.E.M. (D) Pathological changes were also observed in lymphoid tissue. (TIF) [file ppat.1007193.s009.tif]

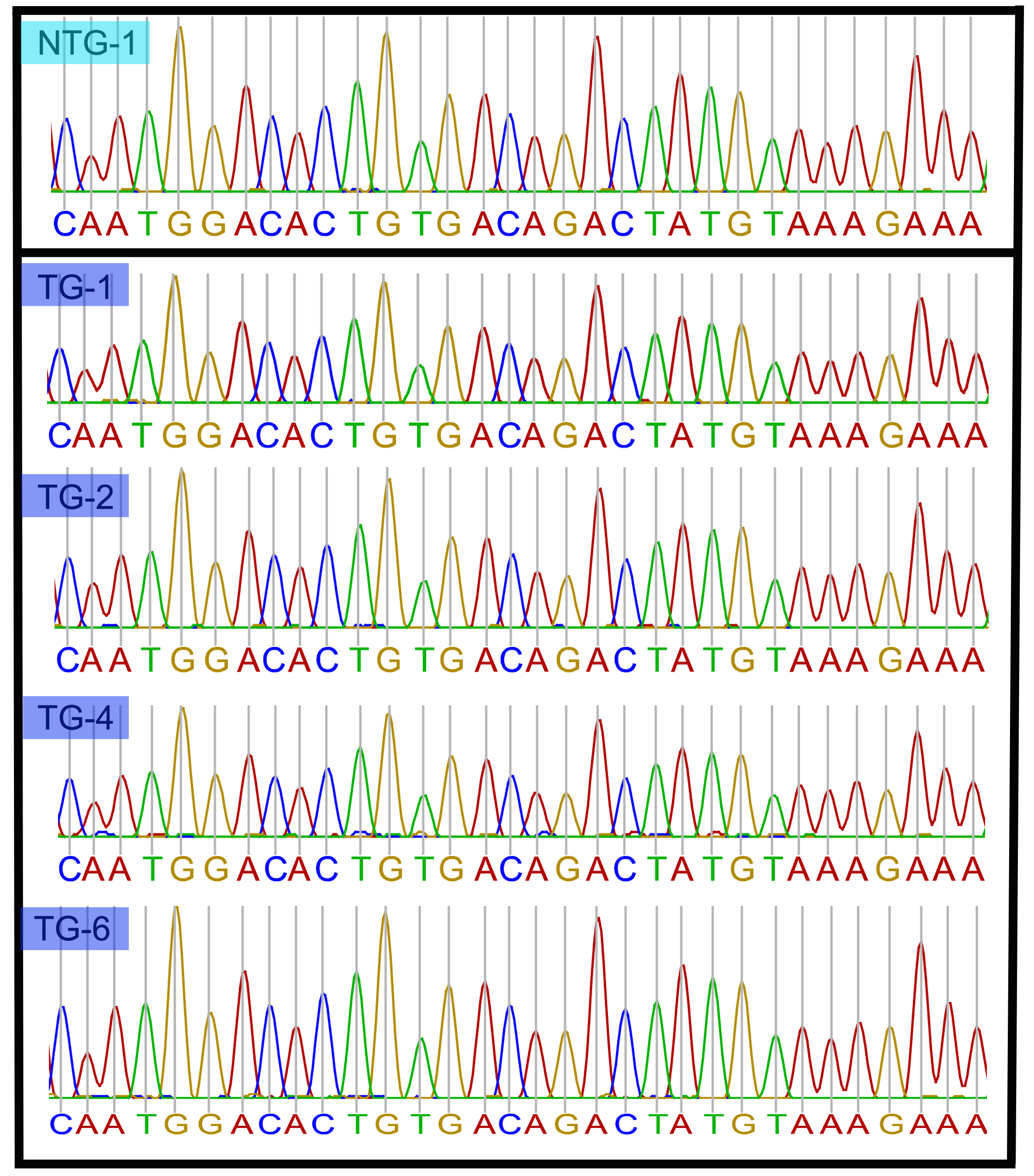

Supplement: S10 Fig — Sequencing results indicated that there was no mutation within the target sequences and flanking regions. NTG: challenged non-transgenic pigs; TG: challenged transgenic pigs. (TIF) [file ppat.1007193.s010.tif]
